# Hold on tight: the kinetic profiling of opioid receptor ligands using the CORAL-MD

**DOI:** 10.1186/s13321-026-01211-8

**Published:** 2026-06-26

**Authors:** Kinga Kurowska, Wiktor Rorat, Szymon K. Kordylewski, Sabina Podlewska

**Affiliations:** 1 Present Address: Maj Institute of Pharmacology, Polish Academy of Sciences, Smętna 12, 31-343 Kraków, Poland; 2 Faculty of Biochemistry, Biophysics and Biotechnology, Jagiellonian University, Gronostajowa 7, 30-387 Kraków, Poland

**Keywords:** μ-opioid receptor, Residence time, Molecular dynamics, Biased signaling, Structure-kinetics relationships, On-line tool

## Abstract

Opioid receptors, particularly the μ-opioid receptor, are central targets in pain management, yet opioid therapeutics remain limited by adverse effects. Recent work indicates that ligand residence time may contribute to signaling bias (a potential avenue to dissociate therapeutic effects from deleterious side effects) by stabilizing distinct receptor conformations. Here, we investigated molecular determinants underlying prolonged residence time of μ-opioid receptor ligands. Seven compounds with experimentally measured residence times and diverse signaling profiles were studied using conventional MD, random accelerated MD, and metadynamics to characterize their unbinding behavior and contact patterns with the receptor.

To enable the systematic extraction and qualitative exploration of interaction features associated with residence time, we developed CORAL-MD (Correlational Analysis of Ligand–Protein Interactions)–an automated platform that analyzes MD-derived interaction profiles in relation to compound kinetics and other experimental outcomes (activity/properties). Application of CORAL-MD revealed specific interaction motifs associated with extended ligand retention, offering valuable guidance for designing safer, more selective opioid therapeutics. The tool is freely accessible at http://coralmd.if-pan.krakow.pl and it facilitates biological interpretation of MD simulations without requiring any programming expertise.

**Scientific contribution**

We identified key ligand-receptor interaction patterns and structural determinants that contribute to prolonged complex stability, including persistent interactions with Asp147^3.32^ and Tyr148^3.33^, as well as additional stabilizing contacts in the extracellular vestibule and at the TM5/TM6 interface. However, the interaction patterns can differ depending on the initial receptor conformation, which should be carefully selected on the basis of the functional effect triggered by the examined compounds. Our findings deepen the mechanistic understanding of binding kinetics in opioid receptors and offer valuable guidance for the rational design of next-generation opioid therapeutics with improved efficacy and reduced side effects. We also share CORAL-MD, an on-line tool (freely-available at http://coralmd.if-pan.krakow.pl), enabling analysis of dependencies between ligand-interaction patterns from molecular dynamics simulations and compound activities, kinetic properties or other experimentally verified features.

**Graphical Abstract:**

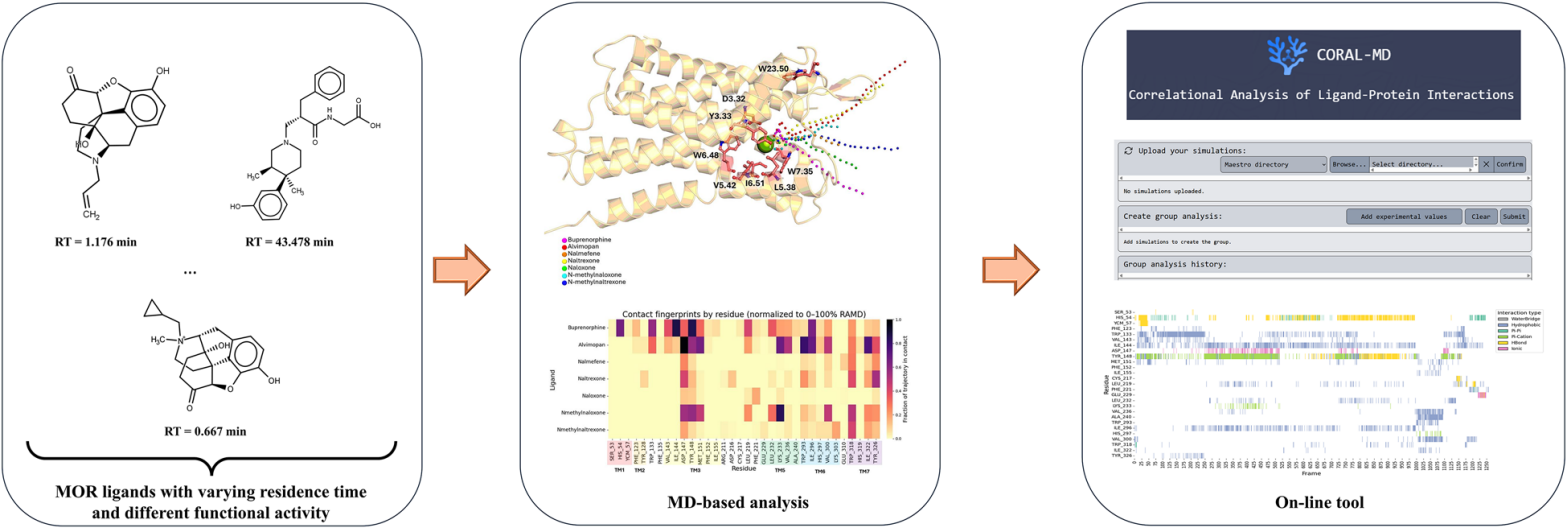

**Supplementary Information:**

The online version contains supplementary material available at 10.1186/s13321-026-01211-8.

## Introduction

Both acute and chronic pain are becoming increasingly widespread globally, creating substantial burdens for healthcare systems [1–3]. Pain not only reduces quality of life but also impairs normal daily functioning [4]. Although existing analgesics, particularly opioids, are effective, their use is limited by the risk of severe side effects, prompting a search for alternative, safer therapeutic approaches [5–8].

Currently, opioid analgesics remain the cornerstone of pain management, with their primary mechanism of action involving activation of the μ-opioid receptor (MOR) [9]. However, conventional opioids are frequently associated with adverse outcomes such as respiratory depression, the development of tolerance, and the potential for addiction. An emerging strategy to address these limitations is the development of MOR-targeting ligands that exploit biased signaling.

Biased (or functionally selective) ligands selectively activate specific intracellular signaling pathways while avoiding others. Increasing evidence suggests that the harmful effects of classical opioids may stem largely from engagement of the β-arrestin signaling cascade. This has led to growing interest in ligands that preferentially activate G protein signaling while minimizing β-arrestin recruitment. Such biased MOR agonists hold promise not only for maintaining effective analgesia but also for reducing the incidence of opioid-related side effects [10–15].

Recently, biased signaling has been increasingly linked to the kinetic properties of ligands, particularly the duration of ligand-receptor complex (residence time, RT). According to this concept, prolonged ligand-receptor engagement can favor stabilization of specific receptor conformations and sustain signaling via particular pathway. These findings suggesting that modulating RT can have impact on functional selectivity open new research avenues for the search of functionally biased MOR ligands [16, 17].

Despite the potential impact of ligand RT on the functional outcomes associated with MOR activation [18–21], the precise molecular determinants responsible for prolonged ligand binding and functional selectivity remain poorly understood. A major challenge lies in the limited availability of experimental RT data for MOR-targeting compounds, which constrains efforts to systematically analyze or model how specific structural moieties affect dissociation kinetics. This data gap not only impedes the development of predictive structure-kinetics relationships but also hampers the rational design of ligands with optimized kinetic and functional profiles.

Additionally, current computational approaches face significant limitations in capturing ligand-receptor dissociation events with sufficient accuracy. While molecular dynamics (MD) simulations represent the gold standard for modeling ligand-receptor complexes and tracking conformational changes over time, standard MD techniques are generally incapable of accessing the long timescales—often milliseconds to seconds-associated with ligand unbinding [22]. As a result, these processes remain beyond the reach of conventional simulations, necessitating the use of enhanced sampling techniques. Methods such as metadynamics (MetaD) [23], accelerated MD (aMD) [24, 25], random accelerated MD (RAMD) [26, 27], Gaussian accelerated MD (GaMD) [28], and weighted ensemble simulations [29, 30] have been developed to overcome these timescale barriers. However, each of these approaches involves trade-offs in terms of accuracy, computational cost, and interpretability, and there is no universally accepted protocol for modeling ligand dissociation at MOR or other G protein-coupled receptors (GPCRs).

Another major challenge lies not only in performing the MD simulations themselves but also in the subsequent analysis and interpretation of the vast amount of data they generate. The raw trajectories, consisting of thousands to millions of conformations of the ligand-receptor complex over time, do not straightforwardly reveal the key determinants of binding stability or dissociation pathways. Identifying crucial intermolecular contacts, monitoring the evolution of the binding site shape, or capturing subtle conformational rearrangements of the receptor often requires extensive post-processing, involving specialized algorithms and statistical methods.

The limitation in the availability of RT data is particularly pronounced in the context of GPCRs. Although several publicly accessible databases—such as KDBI [31], KOFFI [32], BindingDB [33], and PubChem [34]—provide information on ligand–target binding kinetics, these repositories are generally limited in size and contain only sparse data for individual GPCR targets. The growing recognition of the significance of RT in GPCR pharmacology is underscored by recent academic efforts, resulting e.g. in the construction of a literature-based database of GPCR ligand kinetics (GPCR Ligand Kinetics Database), which to our knowledge represents the only existing repository dedicated exclusively to kinetic data for GPCR-targeting compounds [35]. Despite this advancement, the dataset includes only 10 entries related to MOR ligands, highlighting the persistent and critical gap in publicly available RT data for this receptor. This underscores the urgent need for more systematic experimental studies and data curation initiatives focused on ligand kinetics in GPCR systems to enable comprehensive structure-kinetics-function analyses.

In this study, we performed a comprehensive analysis of seven MOR ligands included in the GPCR Ligand Kinetics Database (https://potterton48.github.io/): buprenorphine, alvimopan, nalmefene, naltrexone, naloxone, N-methylnaloxone, and N-methylnaltrexone [35]. These compounds exhibit a wide range of experimentally reported RT, spanning from 0.667 min to 62.5 min, as well as differing functional profiles, including varying degrees of biased signaling at MOR. Given this functional diversity, we aimed to explore potential relationships between ligand structure, dynamic interaction patterns with the receptor, and experimentally determined kinetic and signaling data. Each ligand was subjected to MD simulations using three distinct MOR crystal structures: two representing the inactive state (PDB IDs: 4DKL [36] and 9BJK [37]) and one representing the active state (PDB ID: 5C1M [38]) of the receptor. Ligand-receptor complexes were initially generated through molecular docking, followed by 2000 ns of conventional MD simulation for each system. In addition, selected ligands underwent RAMD and MetaD simulations to model the dissociation process and gain insight into potential kinetic determinants. RAMD was applied to identify dominant ligand egress pathways, which subsequently informed the definition of system-specific collective variables for MetaD simulations within Schrödinger’s unbinding kinetics workflow [39]. Ligand-receptor interaction patterns were analyzed in terms of contact frequency, interaction persistence, and fluctuation of ligand–protein contacts over time. This analysis enabled the identification of specific molecular features and interaction motifs associated with prolonged RT. The results contribute to a deeper understanding of the structural and kinetic factors influencing MOR ligand behavior and may provide valuable guidance for the rational design of next-generation opioid therapeutics with improved efficacy and safety profiles.

In response to the challenges associated with the analysis of MD simulation results, we developed an online tool that facilitates the exploration of dependencies between ligand-receptor contact profiles derived from simulations and experimentally determined features such as activity, kinetic parameters, or other pharmacologically relevant properties. We named it CORAL-MD: Correlational Analysis of Ligand–Protein Interactions and made it freely accessible via an online webpage at: http://coralmd.if-pan.krakow.pl. To the best of our knowledge, it is the first web-based service that facilitates correlational studies between experimental data and multiple MD simulation outputs, thereby supporting automatic structure–activity relationship (SAR), structure-kinetics relationship (SKR), and structure-properties relationship (SPR) analyses. This straightforward approach greatly assists in the evaluation of large sets of simulation results, enabling rapid identification of key contacts and residues that warrant particular attention when designing compounds with improved activity or optimized properties. The CORAL-MD webtool’s intuitive interface allows researchers—including those without programming expertise—to easily perform comprehensive analyses and extract important insights from their data.

## Methods

The list of examined MOR ligands together with the values of their RTs are gathered in Table 1. All compounds, except for alvimopan, display a similar structural core, reflecting their shared morphinan scaffold.

**Table 1:** Compound structures and their reported RTs.

Compound name	Compound structure	RT [min]	Functional activity
Buprenorphine		62.5	Partial agonist
Alvimopan		43.478	Antagonist
Nalfemene		3.448	Antagonist
Naltrexone		2.857	Antagonist
Naloxone		1.176	Inverse agonist
N-methylnaloxone		0.833	Inverse agonist
N-methylnaltrexone		0.667	Antagonist

The table summarized the chemical structures of MOR ligands considered in the study, their functional activity towards MOR and experimentally determined RT reported in the GPCR kinetic database [35] (https://potterton48.github.io/).

The compounds were prepared for docking using LigPrep from the Schrödinger Suite: protonation states were generated at pH 7.4 ± 0.0, and all possible stereoisomers were enumerated; other settings remained at default (throughout the whole study, the 2025_1 version of the software was used). The crystal structures used in the study were fetched from the PDB database and prepared for docking using the Protein Preparation Workflow from the Schrödinger Suite (crystal structures of the following PDB IDs were used: 4DKL, 5C1M, 9BJK). The center of mass of the co-crystallized ligand constituted the grid center in each case, and the grid size was set to 23 Å. The docking was carried out in Glide with default settings. The obtained ligand-receptor complexes were used as an input for MD simulations. They were carried out in Desmond, using the TIP3P solvent model [40], POPC (palmitoyl-oleil-phosphatidylcoline) as a membrane model, the OPLS4 force-field under the pressure of 1.01325 bar, and a temperature of 300 K. The system was solvated in an orthorhombic simulation box with a minimum buffer distance of 10 Å between the solute and the box boundaries. The final box dimensions were automatically determined based on the size of the solute and the specified padding. In each case, the system was neutralized by addition of the appropriate number of Cl^−^ ions and relaxed before simulation; the duration of each simulation was equal to 2000 ns. Protein–ligand interactions were analyzed with in-house Python scripts specifically developed for this study. Ligand dissociation was further investigated using RAMD and MetaD protocols as implemented in the Schrödinger Suite. For RAMD and MetaD, we used docking poses with 4DKL crystal structure, applying default with POPC membrane. Prior to the unbinding simulations, the systems were parameterized with custom charges and relaxed through a multi-step membrane equilibration protocol. This procedure included initial Brownian dynamics at 10 K with heavy atom restraints, followed by stepwise heating to 300 K under NPT/NPgT ensembles with gradual restraint release, and concluded with unrestrained NVT runs. The interactions between ligands and the respective proteins during MD simulations were analyzed using the Simulation Interaction Diagram from the Schrödinger Suite. The geometric criteria used to define individual types of non-covalent interactions are provided below:

- Hydrogen bonds—defined using D–H···A–X geometry with: H···A distance < 2.8 Å, D–H–A angle > 120°, and H···A–X angle > 90°. Interactions are classified as backbone or side-chain and as donor or acceptor.
- Hydrophobic contacts (general)—defined when a hydrophobic protein side-chain atom was within 3.6 Å of a ligand aromatic or aliphatic carbon atom.
- π–π stacking:
- Face-to-face: centroid–centroid distance < 4.4 Å and interplanar angle < 30°,
- Face-to-edge: centroid–centroid distance < 5.5 Å and interplanar angle > 60°.
- Cation–π interactions—defined when the centroid of an aromatic ring and a positively charged group were within 4.5 Å,
- Ionic interactions—defined for oppositely charged ligand and protein atoms within 3.7 Å.
- Water bridges—identified when a water molecule simultaneously formed hydrogen bonds with ligand and protein. For each H-bond: H···A distance < 2.7 Å, D–H–A angle > 110°, and H···A–X angle > 80°. Classified according to protein donor/acceptor role.

For RAMD, the centroid trajectories were analysed, and for MetaD the trajectory with the closest computed residence time to the residence time computed based on all 10 MetaD trajectories for each compound.

### On-line tool

The CORAL-MD web tool was implemented in Python using the Django web framework [41]. To analyze protein–ligand interactions, it uses PLIP [42]. Extracted results are then matched against data from GPCRdb [43] and ChEBI [44] to provide additional information about the supplied trajectory. Several interactive visualizations are generated, using the library Plotly [45]. The interaction priority in our analysis was established based on the typical energetic contribution and directional strength of each contact type. Specifically, we followed a hierarchy where ionic interactions (salt bridges) are prioritized due to their high electrostatic strength, followed by hydrogen bonds and π-interactions (π- π stacking and π-cation), which provide significant stability and orientation. General hydrophobic contacts and water bridges were ranked lower, as they often represent more transient or less energetically dense interactions compared to a direct salt bridge or a strong H-bond. The application is accessible online at http://coralmd.if-pan.krakow.pl without local installation.

## Results and discussion

At physiological pH, the nitrogen atom in the morphinan scaffold becomes protonated, giving rise to a new stereogenic center. Consequently, both stereochemical configurations emerging at this atom were considered in our study. All analyses and correlation studies were carried out independently for three groups of ligands: S isomers, R isomers, and mixed R/S stereoisomers. Additional data not included in the main text are provided in the Supplementary Information; the docking validity was checked via re-docking of the co-crystallized ligand; the comparison of compound orientations in MOR obtained throughout the study is included in the Supporting Information, File S1.

### Analysis of docking results

The orthosteric binding pocket (OBP) of MOR adopts a topology resembling a distorted “Y” shape. High-resolution structural analyses, including cryo-electron microscopy, delineate two principal ligand-binding modes: (i) ligands that occupy a single arm of the “Y,” penetrating deeply into the OBP and projecting toward the extracellular vestibule (e.g., morphine), and (ii) ligands that span both the primary arm and the secondary arm, the latter corresponding to the minor binding pocket [46].

**Fig. 1:**
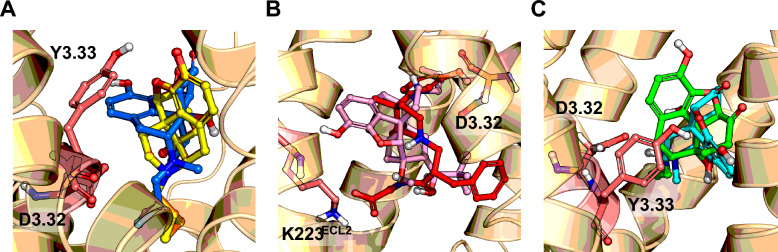
Docking results for compounds with the *S*-configuration at the nitrogen atom obtained for 4DKL-based experiments. The figure presents docking results obtained for one MOR crystal structure (4DKL); remaining data are placed in the Supplementary Information: **A** naltrexone (yellow), nalmefene (orange) and N-methylnaltrexone (navy blue); **B** alvimopan (red) and buprenorphine (magenta); **C** naloxone (green) and N-methylnaloxone (cyan).

In both modes, a conserved salt bridge between the ligand’s tertiary amine and Asp147^3.32^ is observed, a contact recognized as essential MOR pharmacological activity; this interaction was consistently detected across all ligands investigated in the present study (Fig. 1, Supplementary Information Figures S2-S4) [18, 46].

The visual inspection of the obtained docking poses confirms similar fitting of all the compounds in the MOR binding pocket. All compounds were positioned in sufficient proximity to Asp147^3.32^, allowing the basic nitrogen present in each ligand to form a stable ionic interaction with this residue, thereby anchoring the molecules within the binding pocket. Despite the generally similar orientations of the compounds, qualitative differences are evident. For example, naltrexone and N-methylnaloxone are accommodated slightly deeper in the pocket, distinct orientations are also observed for buprenorphine and nalmefene. Such subtle variations in atomic positioning critically determine the possibility of forming hydrogen bonds either with Tyr148^3.33^ or, as in the case of naloxone and N-methylmorphine, with Lys233^5.40^. For nalmefene, naloxone, naltrexone, N-methylnaloxone, and N-methylnaltrexone, docking revealed additional engagement with the “toggle switch” residue His297^6.52^, mirroring that reported for fentanyl in cryo-EM reconstructions [18]. Such an interaction has been hypothesized to underlie the substantially greater potency of fentanyl—by more than an order of magnitude—relative to morphine [47].

However, docking provides only a static snapshot and cannot guarantee that the obtained binding poses are associated with long-term stability, which is crucial for compound behavior and its ability to modulate receptor activity.

For this reason, we performed a detailed MD-based assessment of ligand–protein interactions, enabling us to evaluate the stability and temporal evolution of the observed contacts. Moreover, to systematically interpret the wealth of MD simulation data generated for the MOR ligands, we employed a correlational analysis approach, later formalized in the CORAL-MD platform. This approach allows establishing relationships between ligand-receptor interaction patterns and experimentally determined properties, such as RT.

### Analysis of MD outcome

**Fig. 2:**
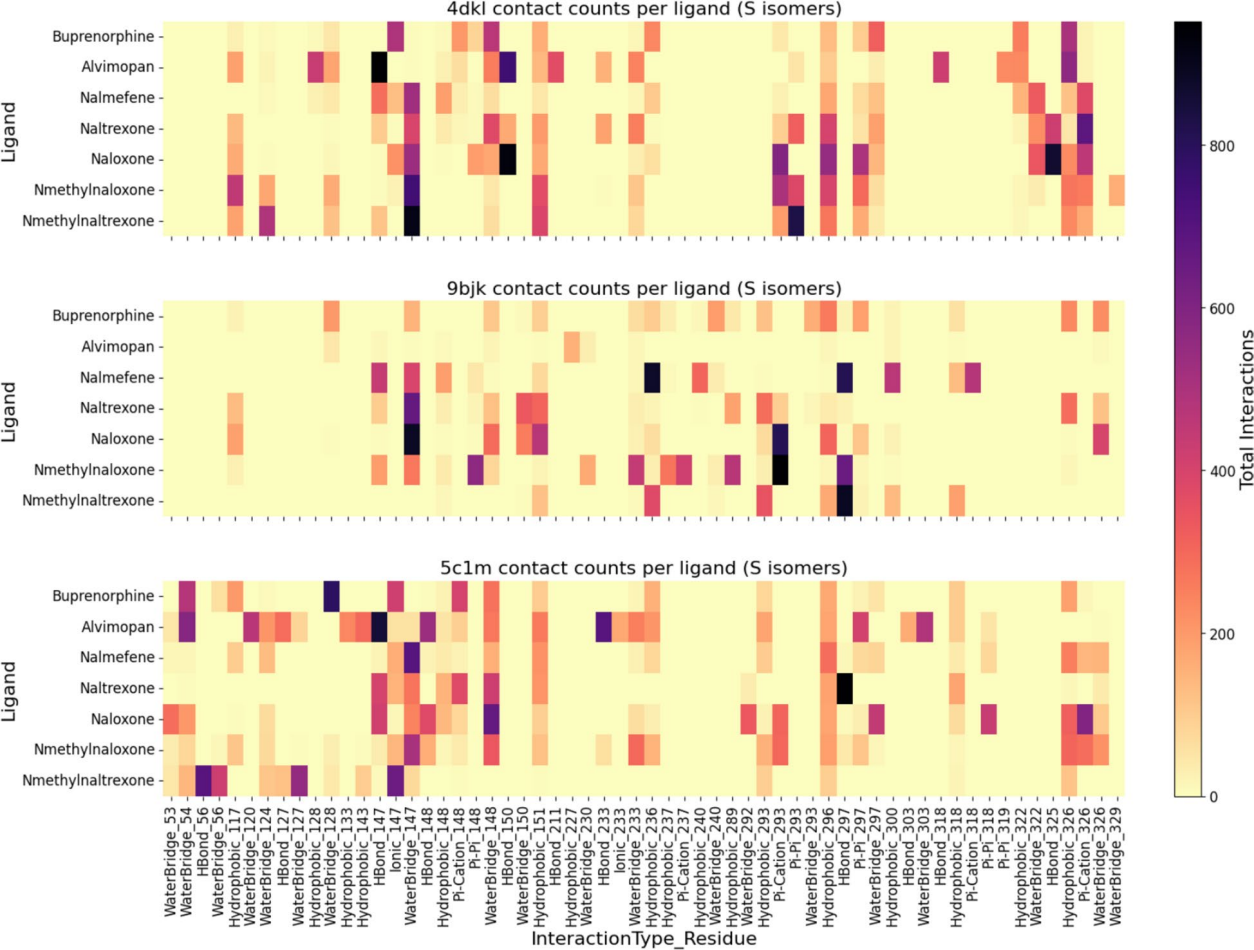
Matrices of ligand–protein contacts showing the distribution of ligand-residue interaction types across three MOR crystal structures. The figure presents contacts of examined ligands (S-isomers) with selected residues for three MOR crystal structures: 4DKL (inactive, antagonist-bound), 9BJK (inactive, inverse agonist-bound), and 5C1M (active, agonist-bound). The y-axis lists the studied ligands and the x-axis corresponds to particular residues for which contacts with ligands were observed, the matrices are colored by the total number of observed contacts during 2000–ns MD trajectories. The figure illustrates that the interaction networks vary substantially between crystal structures, reflecting conformational differences in the receptor.

Each ligand-receptor complex obtained from docking served as the starting point for a 2000 ns MD simulation. Based on these, we generated a dataset quantifying the frequency of specific types of ligand–protein contacts across the simulation time frames (Fig. 2, Table 2). Subsequently, we compared the interaction profiles and RT of each ligand in order to detect molecular determinants for the prolonged receptor occupancy.

**Table 2:** Summary of dominant residue-specific interactions across the three MOR crystal structures considered in the study.

Residue	4DKL (antagonist-bound)	9BJK (inactive, inverse agonist-bound)	5C1M (active, agonist-bound)
53—56	–	–	Water bridges: buprenorphine, alvimopan (mainly with residue 54)
Asp147	Ionic: buprenorphine; H-bond: alvimopan; Water bridges: other ligands (short-RT)	No ionic contacts; H-bond: nalmefene, naltrexone, N-methylnaloxone	Ionic: most ligands (strongest with N-methylnaltrexone; absent with alvimopan, naloxone); H-bond: alvimopan, naloxone, naltrexone; Water bridges: all except alvimopan, buprenorphine, naltrexone
Asn150	H-bond: alvimopan, naloxone	–	–
Trp293	π-cation: naloxone, N-methylnaloxone; π–π: N-methylnaloxone	π-cation: naloxone, N-methylnaloxone	π-cation: naloxone, N-methylnaloxone
His297	π–π: naloxone, N-methylnaloxone (weak); Water bridges: buprenorphine (strong), moderate with other ligands	H-bond: nalmefene, N-methylnaloxone, N-methylnaltrexone	H-bond: naltrexone π–π: alvimopan; Water bridges: naloxone
Ile322	Hydrophobic: buprenorphine, alvimopan, nalmefene; Water bridges: nalmefene, naltrexone, naloxone	–	–
Tyr326	Hydrophobic: buprenorphine, alvimopan; π-cation: short-RT ligands	Nearly absent	Hydrophobic: most ligands (weaker; absent with alvimopan, naltrexone)

The table summarizes the most important ligand–protein contacts for different crystal structures used in the study. It highlights how the same residues adopt different interaction modes depending on the receptor state considered. These comparisons underscore that initial receptor conformation strongly influences which contacts persist.

In this analysis, several positions emerged as critical contributors to the observed interaction patterns and RTs. Three crystal structures were used in the study (two capturing inactive receptor state and one in its active conformation) in order to assess the robustness of the obtained results with respect to the initial conformation. Two inactive conformations were used to evaluate whether the observed ligand-binding behavior remained consistent across receptors representing the same functional state but crystallized with different ligands. In the 4DKL-based studies, where the receptor is preserved in its inactive conformation, Asp147^3.32^ differentiated ligands by interaction type: buprenorphine formed ionic contacts, alvimopan a hydrogen bond, while other ligands engaged with the receptor mainly via water bridges. Tyr326^7.42^ further separated long- and short-RT ligands, with hydrophobic interactions dominating for buprenorphine and alvimopan and cation-π contacts for the short-RT group. Ile322^7.38^ also showed a similar polarity, providing hydrophobic contacts for long-residence ligands but water-mediated interactions for short-residence compounds. Additional contributions included Asn150^3.35^ (hydrogen bonds with alvimopan and naloxone), and the aromatic residues Trp293^6.48^ and His297^6.52^, which supported π-π and cation–π interactions, particularly with naloxone and N-methylnaloxone, alongside water bridges most prominent for buprenorphine.

On the other hand, the 9BJK crystal (also inactive receptor conformation) displayed a contrasting profile of interaction with the examined compounds. Ionic interactions at Asp147^3.32^ were not detected (due to strict geometric criteria for the identification of this contact), with the hydrogen bonds involving nalmefene, naltrexone, and N-methylnaloxone present instead. Tyr326^7.42^ contributed little to the total interaction pattern, while His297^6.52^ played a central role through hydrogen bonding with multiple short-residence ligands. Trp293^6.48^ consistently engaged naloxone and N-methylnaloxone through cation–π contacts, echoing but simplifying the pattern seen in 4DKL. Other residues such as Ile322^7.38^ and Asn150^3.35^ were not involved, marking a sharp departure from the 4DKL interaction landscape.

The experiments based on the activated MOR structure (PDB ID: 5C1M) introduced yet another arrangement. Asp147^3.32^ showed broad but weaker ionic contacts, most frequent with N-methylnaltrexone, and absent with alvimopan and naloxone; hydrogen bonds occurred with alvimopan, naloxone, and naltrexone, while water bridges were widespread except for alvimopan, buprenorphine, and naltrexone. Tyr326^7.42^ retained hydrophobic contributions but less consistently than in 4DKL, and absent for alvimopan and naltrexone. His297^6.52^ engaged ligands through diverse modes, including hydrogen bonds (naltrexone), π-π stacking (alvimopan), and water bridges (naloxone). Notably, residues 53–56 from N-terminus contributed water bridges in this system only, most strongly involving residue number 54 (His) with buprenorphine and alvimopan.

Together, these correlations confirm that distinct residues and interaction types dominate across the three structural templates, further reinforcing that the crystallographic background critically influences which ligand-residue contacts are indicated as responsible for prolonged binding.

Several residues identified in our simulations are positioned within structural elements functionally coupled to the canonical GPCR activation microswitch network [48, 49]. In particular, Trp293^6.48^, forming the core of the conserved toggle switch, and Tyr326^7.53^, located in proximity to the NPxxY motif in transmembrane helix 7, are well-established components of the activation machinery of class A GPCRs. His297^6.52^, situated adjacent to the toggle switch region, may further contribute to the local conformational rearrangements associated with receptor activation.

Comparison of the inactive receptor structures (4DKL, 9BJK) with the active conformation (5C1M) reveals state-dependent differences in contact persistence and interaction geometry of ligands with Trp293^6.48^, His297^6.52^, and Tyr326^7.53^ (Table 2). These findings indicate that the initial receptor conformation substantially reshapes the stabilizing interaction network, particularly within microswitch-coupled regions, and may thereby modulate the energetic barriers governing ligand dissociation.

### Analysis of RAMD and MetaD outcome

**Fig. 3:**
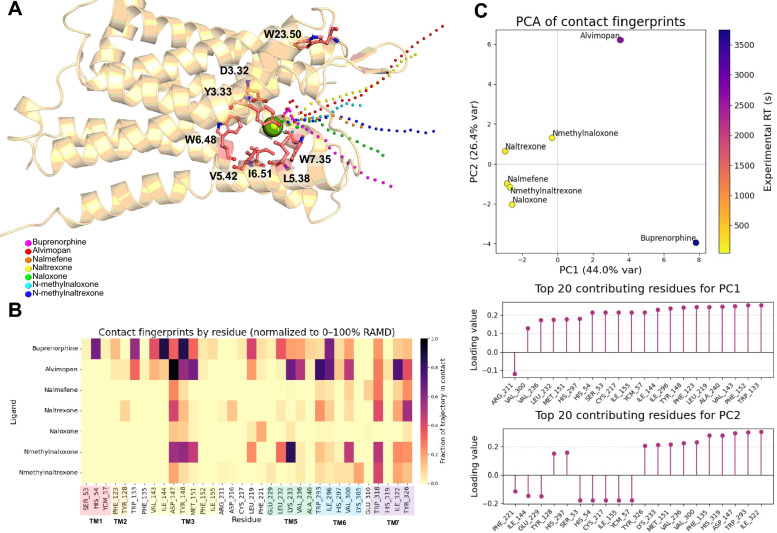
Structural and statistical analysis of ligand dissociation from MOR. The figure presents analysis of RAMD trajectories **A** Representative unbinding pathways obtained from centroid RAMD trajectories. Colored dotted lines indicate dissociation routes for individual ligands, revealing distinct exit directions from the orthosteric pocket toward the extracellular region. **B** Interactions fingerprints for selected residues, showing the fraction of simulation frames in which each ligand maintained contact with a given residue during the unbinding process. Long-RT ligands (buprenorphine, alvimopan) display broader and more persistent contact patterns, particularly involving Asp147^3.32^, Tyr148^3.33^and residues near the TM5/TM6 interface, whereas short-RT antagonists form fewer and more transient interactions. **C** PCA of the contact fingerprints, with color coding by experimental RT. Short-RT ligands cluster together, reflecting similar interaction profiles, while buprenorphine and alvimopan occupy distinct positions: buprenorphine separated mainly along PC1 and alvimopan along PC2—indicating different molecular determinants underlying their extended RT. The accompanying loading plots identify the top 20 residues contributing to each principal component, highlighting residues that dominate the variance and define the separation between short- and long-RT ligands.

**Fig. 4:**
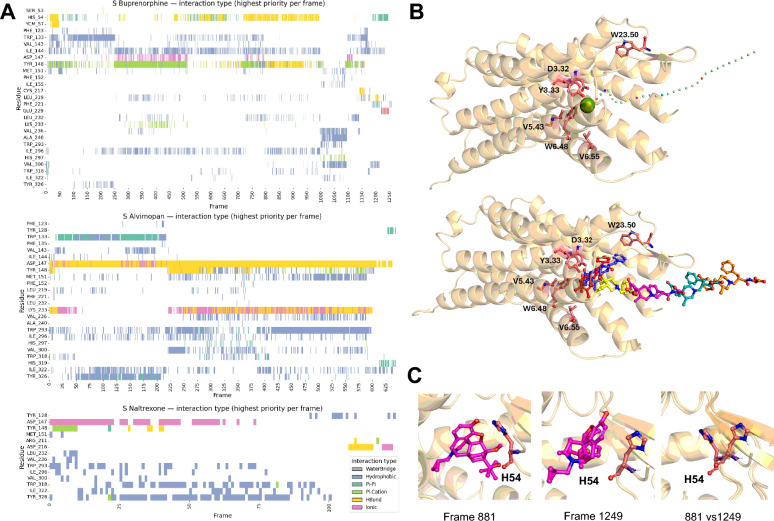
Time-resolved ligand-receptor interactions and unbinding mechanisms observed in RAMD simulations. The figure presents the outcome of RAMD simulations. A) Time-resolved interaction maps showing the dominant (highest-priority) interaction type for each frame in the trajectories of buprenorphine, alvimopan, and naltrexone. Colors correspond to interaction categories (legend on the right). These maps reveal distinct temporal patterns: buprenorphine alternates between contacts with Asp147³·³² and Trp133²·⁵⁰, alvimopan maintains persistent hydrogen bonding to Asp147³·³² and hydrophobic contacts with Trp133²·⁵⁰ and TM5/TM6 residues, while naltrexone rapidly loses its initial ionic contacts and dissociates. Interaction maps of all compounds are shown on Supplementary Figures S5.1-S5.7. B) Representative unbinding pathway of alvimopan derived from the centroid RAMD trajectory. The upper panel shows the dissociation route as colored dots, corresponding to the ligand conformations shown below. Key residues along the pathway - mark the main interaction sites during unbinding. C) Conformational changes of His54 observed in the buprenorphine trajectory (5C1M structure) at frames 881 and 1249. The residue shifts position as the ligand transitions from the orthosteric pocket toward the extracellular region, indicating a gating-like role that transiently hinders ligand exit.

Thanks to RAMD and MetaD simulations, we were able to predict distinct escape pathways of the ligands. The example analysis of their dissociation trajectories and potential exit routes is presented in Fig. 3 A. Furthermore, detailed analysis of ligand-protein contacts observed during dissociation simulations performed in Desmond (Fig 4) provided estimated RTs, which we later confronted with the experimental values.

### Time-resolved interaction patterns during RAMD trajectories

Principal component analysis (PCA) of the contact fingerprints revealed clear separation of ligands by their RTs, with PC1 accounting for most of the variance (44.0%). Buprenorphine, the ligand with the longest experimental RT, formed a distinct cluster on the positive side of PC1, reflecting a unique interaction pattern compared to the short-RT antagonists. Alvimopan, another long-RT ligand, was also separated from the antagonist cluster, mostly along PC2, underscoring differences in its binding contacts. In contrast, the short-RT ligands (e.g., naltrexone, naloxone, nalmefene, N-methylnaloxone, N-methylnaltrexone) clustered together, indicating similar contact profiles during unbinding (Fig. 3 C).

Time-resolved analysis of RAMD trajectories further highlighted these differences. Across all ligands, the early phase of the RAMD trajectories was characterized by consistent interactions with three key residues in the binding pocket: Tyr148^3.33^, Asp147^3.32^, and Tyr326^7.42^. Tyr326^7.42^ formed primarily hydrophobic contacts, occasionally supplemented by π–cation interactions, while Tyr148^3.33^ engaged in diverse interactions, including hydrogen bonds, π–π stacking, π–cation, and hydrophobic contacts. Asp147^3.32^ was particularly critical: in short-RT ligands (e.g., naltrexone, naloxone, N-methylnaltrexone), this residue was stabilized mainly through ionic interactions, which tended to disappear within the first third to half of the trajectory, coinciding with ligand dissociation.

In contrast, alvimopan, one of the ligands with long RT, exhibited a markedly different pattern. Instead of transient ionic contacts, Asp147^3.32^ was engaged in stable hydrogen bonds that persisted throughout almost the entire simulation, providing a continuous anchor point. Uniquely, in the early phase, alvimopan also displayed π–π and hydrophobic interactions with Trp133^23.50^ (ECL1), which were absent in short-RT ligands. Furthermore, alvimopan showed a distinctive intermediate-phase interaction pattern, maintained well beyond the point where other ligands disengaged. This involved residues on the TM5/TM6 interface, including Val236^5.43^ and Val300^6.55^ (hydrophobic contacts), Lys233^5.40^ (ionic and hydrogen bonding), and Trp293^6.48^ (hydrogen bonding and occasional π-π stacking). Tyr148^3.33^ also remained engaged for a prolonged period in alvimopan compared to short-RT ligands.

For buprenorphine, the interaction profile showed several distinctive features compared to both long- and short-RT antagonists. Similar to alvimopan, it engaged Trp133^23.50^ through hydrophobic contacts, but unlike alvimopan this interaction alternated with bonding to Asp147^3.32^ rather than occurring simultaneously, suggesting a more dynamic switching mechanism. In addition, buprenorphine formed hydrogen bonds and π-π interactions with His54, located at the extracellular pocket entrance, which likely acted as a gate slowing ligand egress. However, this interaction could not be noticed in different ligands, as 4DKL crystal structure starts with Met65. The trajectory further revealed stable hydrophobic contacts with Val143^3.28^ and Ile144^3.29^, residues that were only weakly engaged by alvimopan and absent in short-RT ligands.

A common feature of the long-RT ligands was engagement of Trp133^23.50^, located in ECL1, together with interactions at Asp147^3.32^, a residue critical for anchoring ligands in the binding pocket. In alvimopan, Trp133^23.50^ contacts occurred concurrently with persistent hydrogen bonding to Asp147^3.32^, complemented by the formation of a secondary interaction cluster on the TM5/TM6 interface, which together stabilized the complex over the full trajectory. In buprenorphine, by contrast, Trp133^23.50^ interactions alternated with hydrogen bonds to Asp147^3.32^, while additional stabilization was provided by hydrogen bonding and π-π stacking with His54 at the extracellular entrance and hydrophobic contacts with Val143^3.28^/Ile144^3.29^. Short-RT ligands, in turn, rapidly lost their initial ionic contacts to Asp147^3.32^ and failed to establish compensatory interactions, resulting in fast dissociation.

### Infrequent MetaD simulations and identification of metastable states

**Fig. 5:**
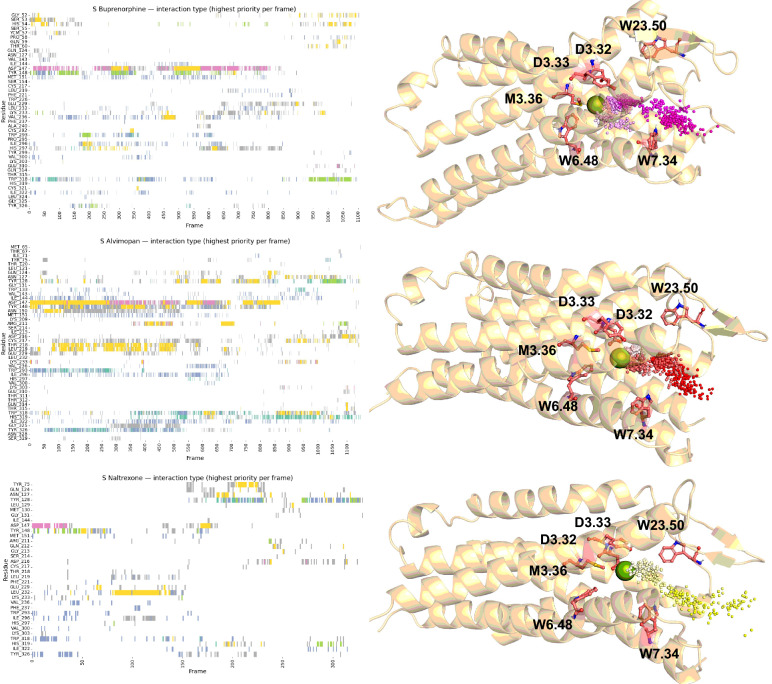
Representative ligand dissociation trajectories obtained from MetaD simulations. The figure presents results obtained in the MetaD simulations: Left panels: Time-resolved interaction maps for buprenorphine, alvimopan, and naltrexone, showing the dominant (highest-priority) interaction type per frame. Colors correspond to interaction categories (legend as in Fig. 4A). Interaction maps of all compounds are shown on Supplementary Figures S6.1-S6.7. Right panels: 3D representations of the receptor with the corresponding ligand dissociation pathways obtained in MetaD simulations. The color gradient along each trajectory indicates simulation time (darker colors correspond to later frames). Key residues shaping the unbinding routes are highlighted.

Based on the RAMD centroid trajectories, we performed infrequent MetaD simulations to estimate ligand RT. This method applies minimal bias during unbinding and reweights the biased simulation time to recover physical dissociation times. The resulting RT values are shown in the next subsection, and representative trajectories are shown in Fig. 5.

**Fig. 6:**
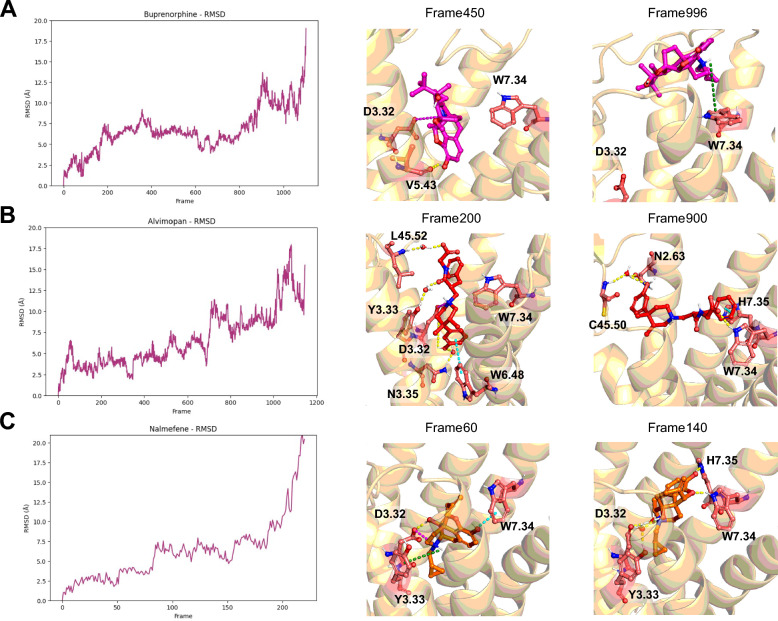
Ligand–protein RMSD profiles and representative metastable states identified in MetaD trajectories. The figure presents RMSD plots for buprenorphine **(A)**, alvimopan **(B)**, and nalmefene **(C)** and it shows two characteristic metastable states corresponding to distinct stages of ligand dissociation. It was determined in such a way that the protein–ligand complex was first aligned on the protein backbone of the reference and then the RMSD of the ligand heavy atoms was measured. The lower RMSD plateau represents the ligand confined in the orthosteric binding pocket, while the higher plateau corresponds to the intermediate state located near the extracellular vestibule. For buprenorphine and alvimopan, these metastable states persist over several hundred frames, indicating long-lived intermediate binding. In contrast, for the short-RT ligand nalmefene, the second state is short-lived and quickly transitions to full dissociation. Representative frames illustrate key interactions stabilizing these states, involving Asp147^3.32^ and Tyr148^3.33^ within the orthosteric site, and His^7.35^ or Trp318^7.34^ in the vestibular region, which transiently anchor the ligand before exit. RAMD plots of all compounds and interaction diagrams of metastable poses are shown on Supplementary Figures S6-S7.

Analysis of ligand–protein RMSD profiles (Fig. 6) revealed the presence of two metastable states for most ligands: one in the orthosteric binding pocket and a second in the vestibule region, located between the pocket and the extracellular side. This vestibule likely contributes to receptor modulation and may influence signaling outcomes [15]. In short-RT ligands, the vestibular state was unstable or short-lived (~ 50 frames), whereas in long-RT ligands it persisted for 200–300 frames. The key residues stabilizing ligands in this region were Trp318^7.34^ and His319^7.35^, which formed π-π and π-cation interactions, respectively.

### Relationship between experimental and computed RT

**Fig. 7:**
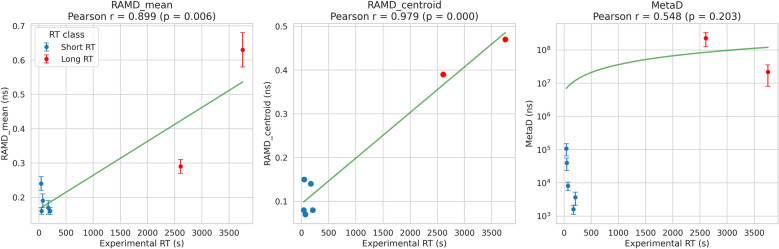
Comparison of experimental and computed RT from RAMD and MetaD simulations. The figures presents correlation between the experimental and predicted RT values. From left to right: mean RAMD RT, RAMD centroid RT, and MetaD-derived RTs. Mean RAMD values show moderate correlation with experiment (r = 0.876) and limited separation between short- and long-RT ligands. RAMD centroid simulation times performs best (r = 0.979), providing clear distinction of buprenorphine and alvimopan from the short-end period RT group. MetaD shows weaker linear correlation (r = 0.548) but distinctly separates long- and short-RT ligands across a wide dynamic range.

To assess the predictive performance of the computational approaches, we compared the experimentally determined RTs with values obtained from RAMD (mean simulation time and centroid simulations time), and MetaD simulations (Table 3). Scatter plots of experimental versus computed RTs are shown in Fig. 7, with both linear and logarithmic axes to capture the broad dynamic range of the data.

**Table 3:** Experimental and computed RTs of MOR ligands considered in the study.

Ligand	RT exp (s)	RAMD mean (ns)	RAMD centroid (ns)	MetaD (s)
Buprenorphine	3750	0.63 ± 0.05	0.47	2.19 × 10^1^ ± 1.39 × 10^1^
Alvimopan	2608.68	0.29 ± 0.02	0.39	2.29 × 10^2^ ± 1.04 × 10^2^
Nalmefene	206.88	0.16 ± 0.01	0.08	3.69 × 10^−3^ ± 1.55 × 10^−3^
Naltrexone	171.42	0.17 ± 0.02	0.14	1.60 × 10^−3^ ± 4.78 × 10^−4^
Naloxone	70.56	0.19 ± 0.02	0.07	8.19 × 10^−3^ ± 2.35 × 10^−3^
N-methylnaloxone	49.98	0.16 ± 0.01	0.15	3.99 × 10^−2^ ± 1.64 × 10^−2^
N-methylnaltrexone	40.02	0.24 ± 0.02	0.08	1.08 × 10^−1^ ± 4.32 × 10^−2^

The table compares the experimental RT values (provided in seconds) with the RT estimations obtained via different computational approaches–RAMD (taking into account mean value and value referring to the centroid after data clustering) and MetaD.

For RAMD cluster 0 trajectories mean times, the correlation with experiment was moderate (Pearson r = 0.876), but the separation between long- and short-RT ligands was not clear. In contrast, RAMD centroid-based values performed substantially better: they not only produced an excellent linear correlation with experimental RTs (Pearson r = 0.979), but also provided a more clean separation of long-RT ligands (buprenorphine, alvimopan) from the short-RT group.

MetaD showed weaker linear correlation (Pearson r = 0.548). Nevertheless, the dynamic range of the computed values was sufficient to very clearly distinguish the long-RT ligands from the short-RT cluster, even if the internal ranking within the short-RT set was not captured.

Overall, these results demonstrate that both computational approaches, particularly MetaD, effectively discriminate between long- and short-RT ligands. However, while the methods provide a clear binary separation, they lack the resolution to maintain a consistent ranking for ligands with closely similar experimental values. Consequently, while these tools are powerful for categorical classification, they do not yet allow for the precise numerical prediction of RT across the entire dynamic range.

The success of the utilized computational approaches (RAMD and MetaD via Schrödinger’s unbinding kinetics workflow) in capturing the correct experimental RT ranking stems from their ability to identify and bias the most physically relevant unbinding pathway. Specifically, RAMD generates an ensemble of unbinding trajectories, and the most frequently sampled route is postulated to represent the lowest free energy path. By using this main egress path to define system-specific CVs for the subsequent MetaD step, the simulations accurately accelerate the relevant degrees of freedom for each specific ligand, preserving the relative energetic barriers and thus the rank ordering. Regarding their general reliability, recent comprehensive benchmarks of this exact automated protocol demonstrate its robust predictive power across diverse targets (achieving an R^2^ of 0.80 and RMSE of 1.22 in log10(RT)) without requiring extensive empirical calibration [39]. Therefore, these methods are considered highly reliable for categorical classification and lead prioritization, provided that the unbinding process does not involve major protein backbone rearrangements that are unaccounted for by the ligand-centric CVs.

### Analysis of classic MD simulations

#### Monitoring the R165-Y252 distance

**Fig. 8:**
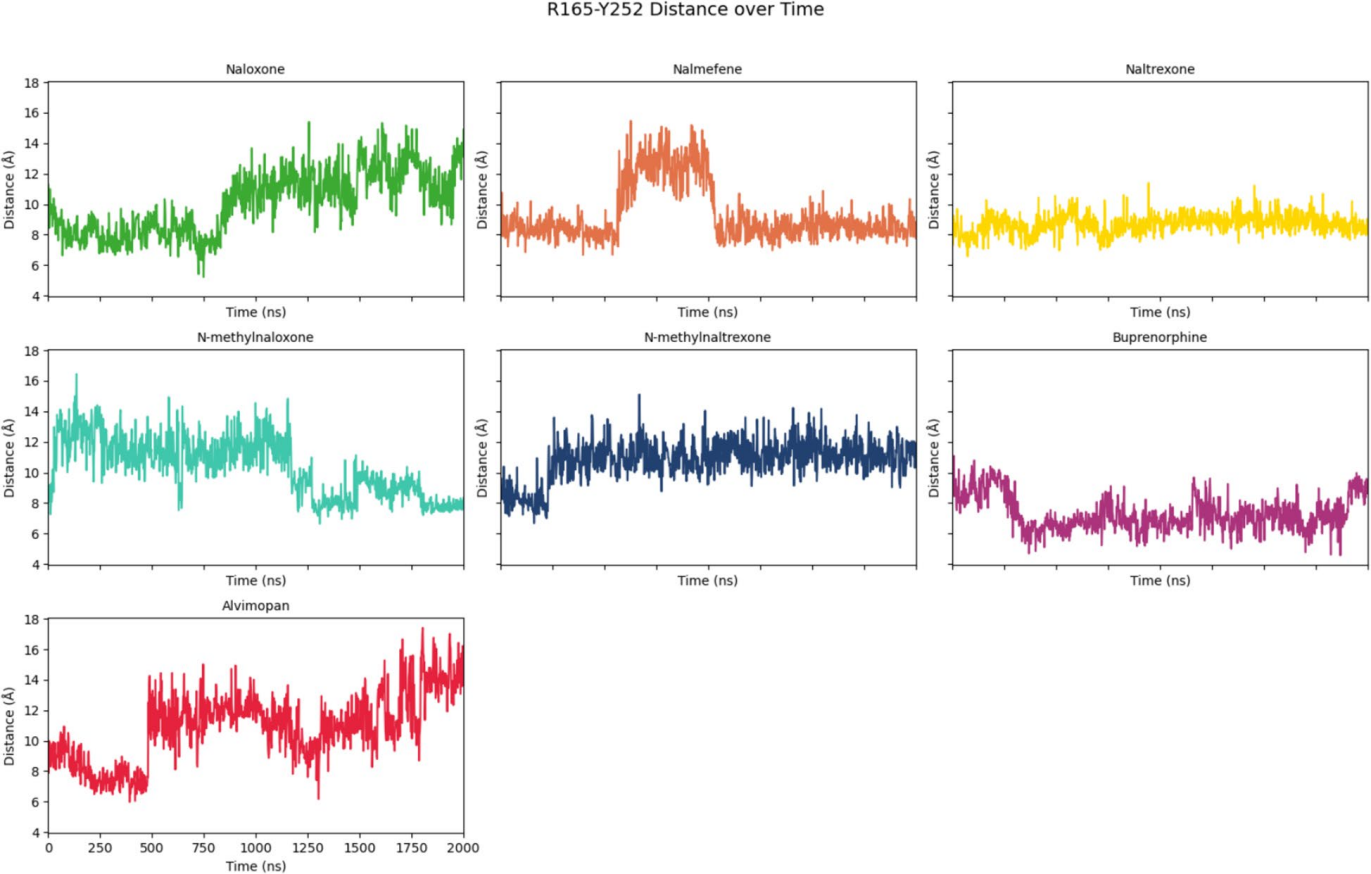
Analysis of R165-Y252 distance observed during MD simulations carried out for the 4DKL crystal structure. The figure presents time evolution of the R165-Y252 distance during MD simulations with different MOR ligands. Antagonists show stable or increased distances, consistent with loss of the hydrogen bond and inactive conformations. In contrast, buprenorphine reduces the R165-Y252 distance toward ~4 Å, compatible with hydrogen bond formation and partial stabilization of the active state.

To assess whether the receptor maintained conformational responses consistent with ligand efficacy during our simulations, we monitored the distance between R165^3.50^ and Y252^5.58^, a contact known to be critical for stabilizing the active MOR state (Fig. 8. Previous experimental studies have shown that the hydrogen bond between the homologous residues R165^3.50^ and Y254^5.58^ contributes to maintaining the outward-shifted conformation of TM6, which is characteristic of the activated receptor state [50].

In our trajectories, antagonists and inverse agonists (naloxone, naltrexone, nalmefene, N-methylnaltrexone, and N-methylnaloxone) displayed either a stable or increasing R165-Y252 distance, consistent with disruption of this hydrogen bond and stabilization of inactive conformations. By contrast, buprenorphine, a partial agonist, drove the distance towards ~ 4 Å, close to the range required for hydrogen bond formation, suggesting partial stabilization of the outward-shifted TM6. In 5C1M and 9BJK, R165-Y252 distance remained stable during most simulations (~ 5 Å in 5C1M and ~ 7.5 Å in 9BJK, Supplementary Figure S9, Figure S10).

Together, these results indicate that the simulations with 4DKL crystal structure reproduce expected functional distinctions: antagonists prevent or disrupt the R165-Y252 contact, whereas buprenorphine promotes its re-formation, in line with its partial agonist profile.

The RMSD values relative to the initial docking poses monitored over MD simulations are presented in the Supporting Information, Figure S11.1-S11.3).

### Analysis of MD trajectories with CORAL-MD

**Fig. 9:**
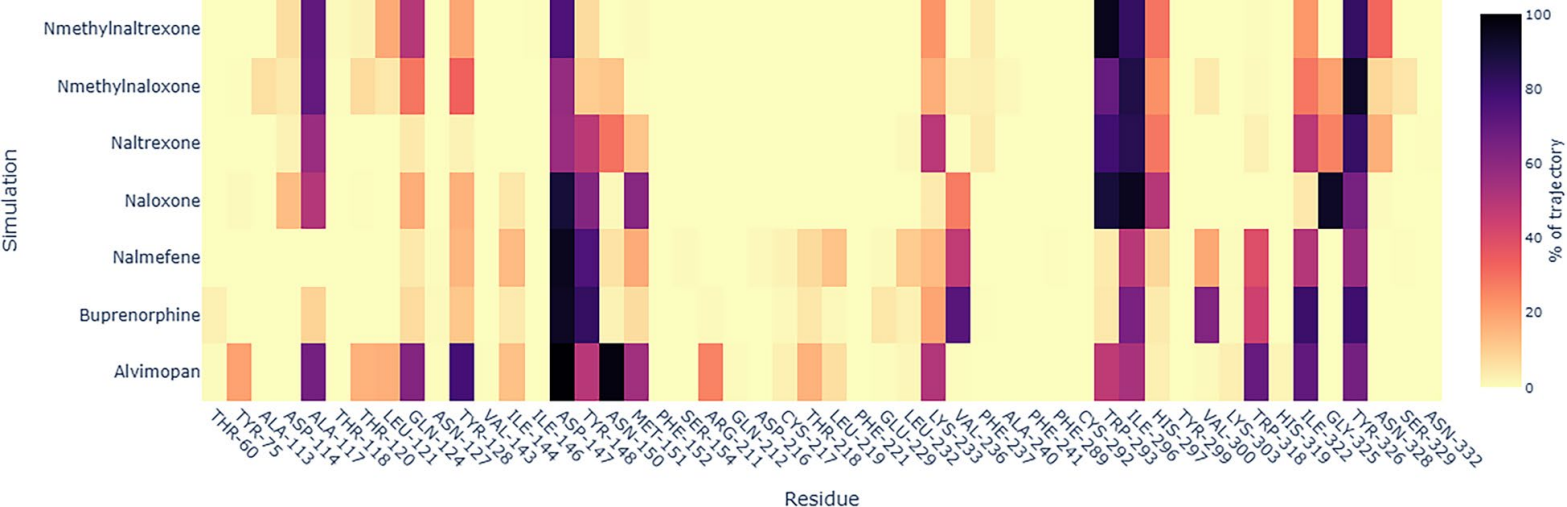
Matrix of ligand-protein interaction occurrence for selected amino acids. The figure shows ligand-protein interaction for selected residues in the form of a matrix, which presents the fraction of simulation frames in which each ligand maintained contact with a given amino acid during the MD simulation. Generated using CORAL-MD webtool for 4DKL-based simulations.

**Fig. 10:**
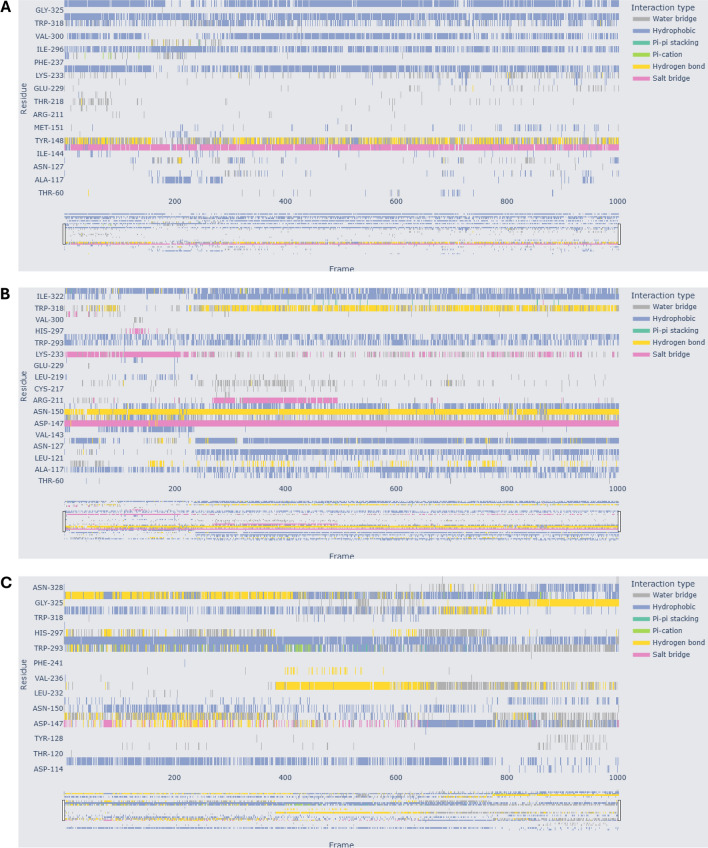
Time-resolved interaction maps for A) buprenorphine, B) alvimopan, and C) naltrexone. The figure shows the dominant (highest-priority) interaction type per frame. Interaction maps of all compounds are shown on Supplementary Figures S12.1-S12.7.

The systematic characterization of ligand-receptor interaction patterns during classical MD simulations was carried out with the use of the CORAL-MD platform (the results discussed in this section are based on simulations carried out for 4DKL crystal structure; Fig.9. This approach enabled time-resolved quantification of residue-specific contacts and direct comparison of interaction persistence across ligands with distinct RTs and the MOR systems analyzed here serve as representative case studies of the platform’s analytical capabilities (Fig.10).

In the classical MD simulations, all ligands maintained stable contacts with Tyr326^7.42^·^42^, Ile296^6.50^⁶·^50⁰^, Trp193^4.50^^4·50⁰^, Asp127^2·5⁰^, and Tyr148^3.33^^3·33^. Among these, Tyr148^3.33^^3·.33^ showed a clear distinction: alvimopan and buprenorphine formed persistent hydrogen bonds, whereas in the short-RT ligands these interactions were weaker or replaced by hydrophobic and water-mediated contacts. A similar pattern was observed for Asp147^3.32^^3·.32^, where alvimopan and buprenorphine displayed strong, long-lasting ionic interactions (99.6% and 89.8% of the trajectory, respectively), while the remaining ligands showed weaker or intermittent engagement, often shifting toward hydrophobic or water-bridge contacts. Both long-RT ligands and nalmefene additionally interacted hydrophobically with Trp318^7.35^⁷^·.35^, a feature absent in the other compounds.

In contrast, short-RT ligands established stable π-π, π-cation, and hydrogen-bond interactions with His297^6.52^, whereas long-RT ligands engaged this residue primarily through weaker hydrophobic contacts. A similar trend was observed for Gly325^7.41^, which formed stable hydrogen bonds only in the short-RT group (with the exception of nalmefene). Unique to alvimopan were hydrophobic contacts with Tyr128^2.64^ and a stable hydrogen bond with Asn150^3.35^, consistent with the MetaD trajectory.

Overall, long-RT ligands were characterized by strong, persistent interactions with key anchoring residues (Asp147^3.32^ and Tyr148^3.33^), while short-RT compounds relied more on transient hydrogen bonds and alternative hydrophobic or water-mediated contacts.

### Coral-MD

**Fig. 11:**
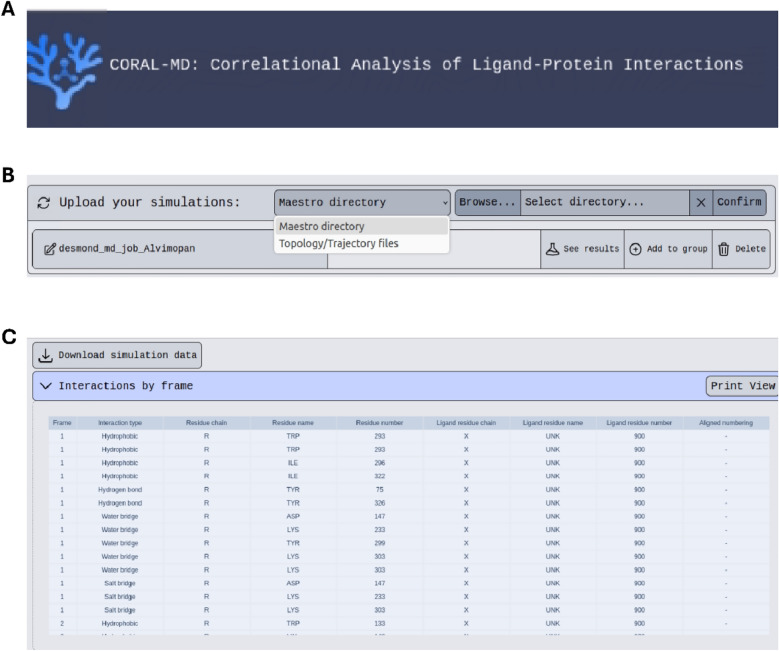
Presentation of CORAL-MD – an on-line tool for MD results analysis. The figure shows the on-line tool constructed within the study: A) logo of the tool, B) process of files submission; VMD-compatible trajectories are supported, C) text file with ligand-protein interactions summary, which can be downloaded as the csv file.

To enable both the systematic analysis of MD-derived ligand-receptor interaction data presented in the preceding sections and broad accessibility of these analytical capabilities to the wider scientific community, we developed CORAL-MD, an online platform designed to perform such analyses quickly, intuitively, and without requiring programming expertise (available at https://coralmd.if-pan.krakow.pl; Fig.11  A). The tool automatically detects and characterizes the principal types of noncovalent interactions, including hydrogen bonds, hydrophobic contacts, and aromatic stacking, and presents them through intuitive, time-resolved visualizations. CORAL-MD supports the upload of single or multiple trajectories, enabling both individual trajectory analysis and comparative, group-based evaluation across different ligands or receptor states. In addition, users may optionally incorporate experimental data, allowing direct assessment of correlations between simulation-derived interaction profiles and measured biological or pharmacological parameters; the scope of the platform extends well beyond the specific receptor system presented here and that it is broadly applicable to diverse ligand–protein systems and MD datasets.

**Fig. 12:**
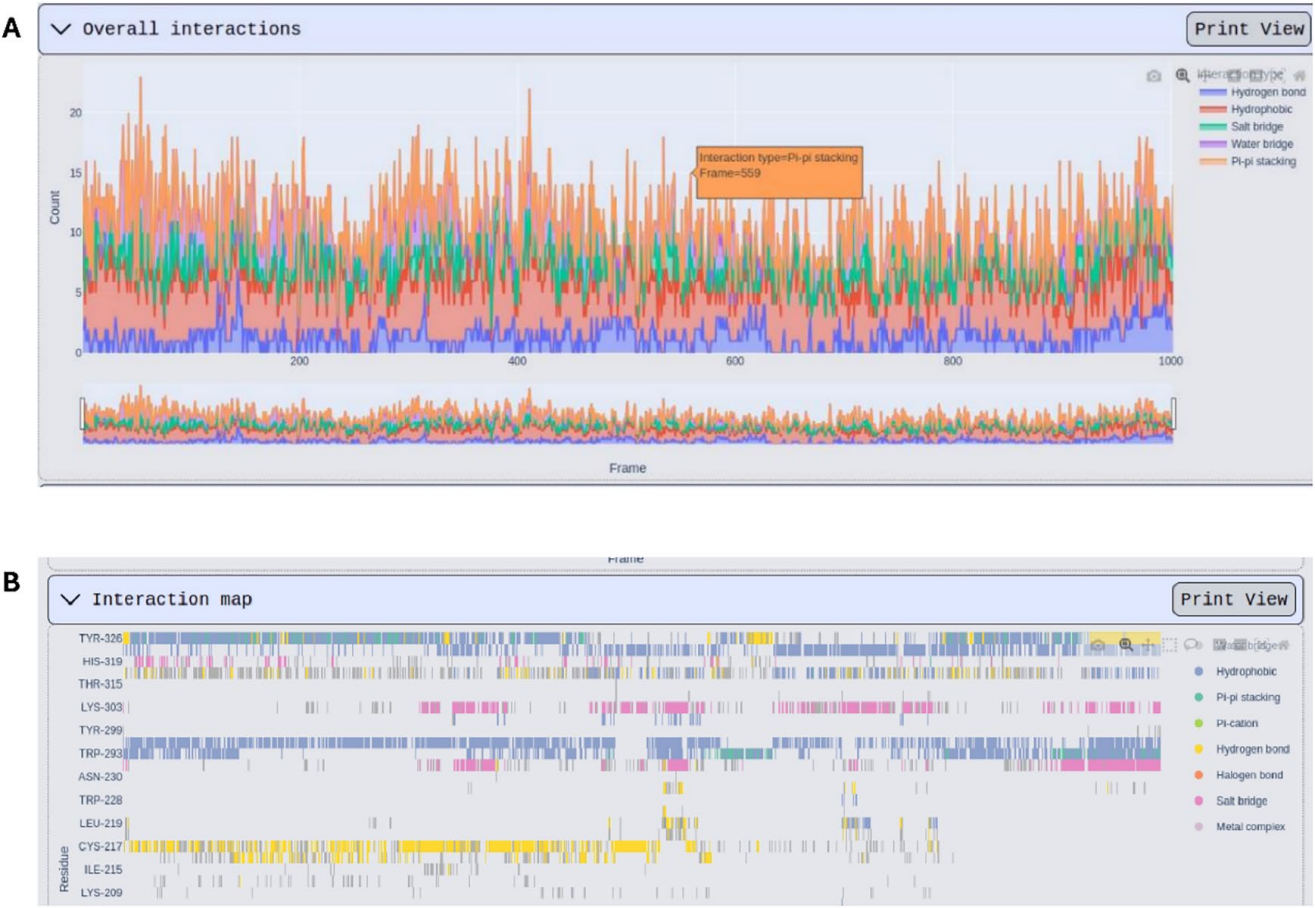
CORAL-MD - analysis of single simulation. The figure shows the analysis of single example MD simulation using CORAL-MD; A) Distribution of particular contacts, B) ligand-protein interaction matrix.

CORAL-MD supports VMD-compatible trajectory formats (Desmond and GROMACS output in particular). Desmond simulations are processed by uploading the entire trajectory directory, whereas GROMACS trajectories require the corresponding topology and trajectory files (Fig. 11 B). For single-trajectory analyses, the tool reports the full history of ligand–protein contacts and visualizes changes in interaction patterns throughout the simulation. Users may download raw contact data in CSV format for external processing (Fig. 11 C) or explore interactive charts in which specific interaction types can be displayed or hidden (Fig.12 A). The tool also generates ligand–protein contact matrices that can be filtered to focus on particular interaction classes (Fig.12  B).

**Fig. 13:**
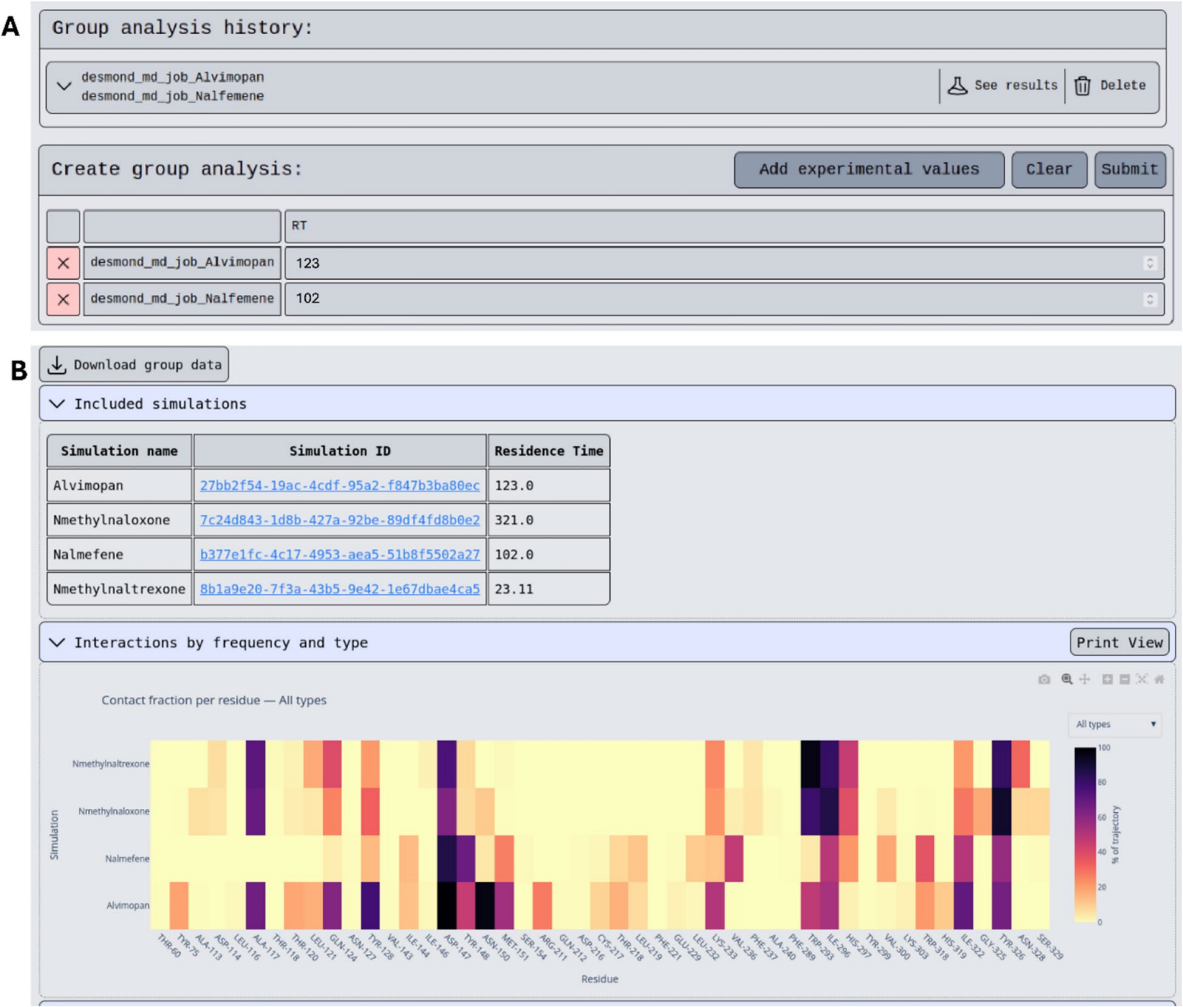
CORAL-MD – group analysis. The figure shows the group analysis of several MD simulations using CORAL-MD; A) adding particular simulations to the group analysis, B) matrix of the frequency of ligand-protein contacts with particular amino acid during simulation.

Batch processing enables comparison of multiple simulations and correlation of ligand–protein contacts with experimental readouts (Fig.13 A). Group analyses highlight differences in the frequency of specific interactions across simulations, facilitating the identification of key contact patterns associated with particular experimental values (Fig 13. B). All results can be exported in several formats for downstream analysis. CORAL-MD is freely accessible without registration or usage restrictions.

## Conclusions

Our study identifies key interaction patterns that govern ligand RT at MOR. Long-RT ligands, such as alvimopan and buprenorphine, maintained persistent engagement with Asp147^3.32^ and Tyr148^3.33^ and formed additional stabilizing contacts in the extracellular vestibule (e.g., Trp133^23.50^) or at the TM5/TM6 interface. These extended interaction networks effectively slowed ligand dissociation, whereas short-RT ligands rapidly lost their initial anchoring interactions and failed to establish compensatory contacts.

The results highlight that prolonged RT arises from both strong primary binding-site interactions and the ability to form transient, sequential interactions along the unbinding pathway.

Finally, we developed CORAL-MD, a freely accessible online platform enabling automated extraction, visualization, and correlation of ligand–protein interaction patterns from MD simulations. By allowing users to relate MD-derived interaction signatures to kinetic or functional data, CORAL-MD facilitates broader application of our analytical approach and supports the medicinal chemistry community in designing next-generation of ligands with optimized properties.

## Supplementary Information

Supplementary Material 1.

## Data Availability

All data used in the study are available in the Supplementary Information. The source code of our online tool is freely available under the GNU GPLv2 license at: https://github.com/metro-maniana/CORAL-MD.

## Abbreviations

aMD: Accelerated MD
ECL: Extracellural loop
GaMD: Gaussian accelerated MD
GPCR: G-protein coupled receptor
MD: Molecular dynamics
MOR: μ-Opioid receptor
OBP: Orthosteric binding pocket
PCA: Principal component analysis
POPC: Palmitoyl-oleil-phosphatidylcoline
RAMD: Random accelerated molecular dynamics
RT: Residence time
TM: Transmembrane
