# Supplementary material for: Hold on tight: the kinetic profiling of opioid receptor ligands using the CORAL-MD
Source: J Cheminform. 2026 Jun 26;18:99. doi: 10.1186/s13321-026-01211-8 (PMC13378134; doi:10.1186/s13321-026-01211-8)
Supplement: Supplementary file 1 — Supplementary Material 1. [file 13321_2026_1211_MOESM1_ESM.pdf]

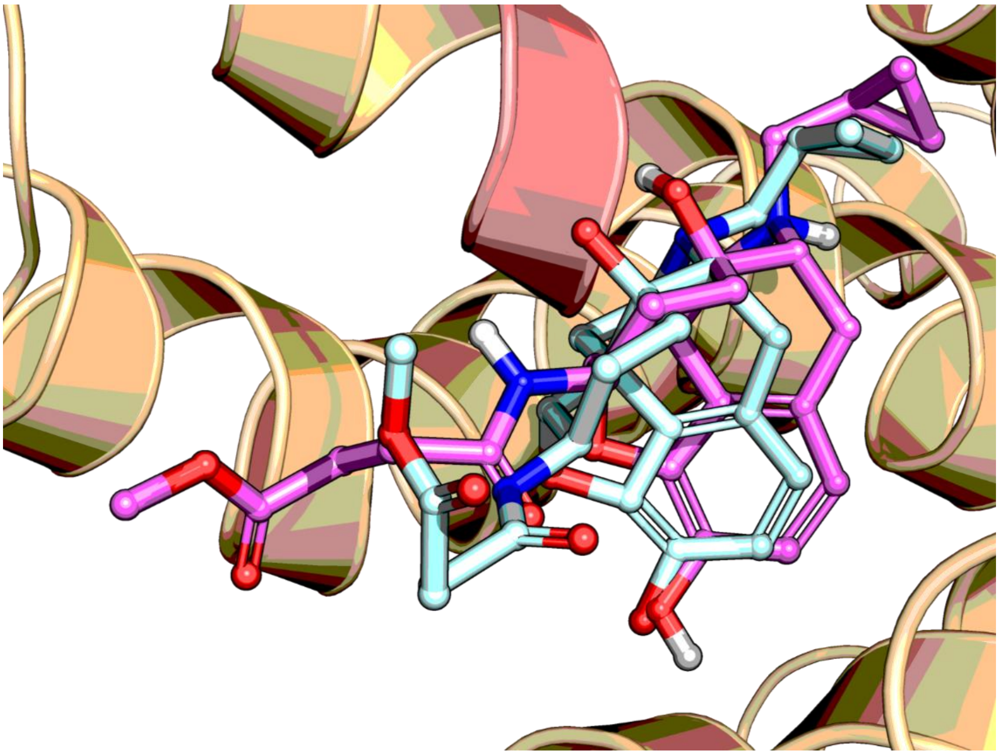

Figure S1. Comparison of docking poses obtained for co-crystallized ligand for 4DKL to confirm the validity of the obtained docking results (co-crystallized ligand: cyan; docked pose: pink), RMSD = 1.576 Å.

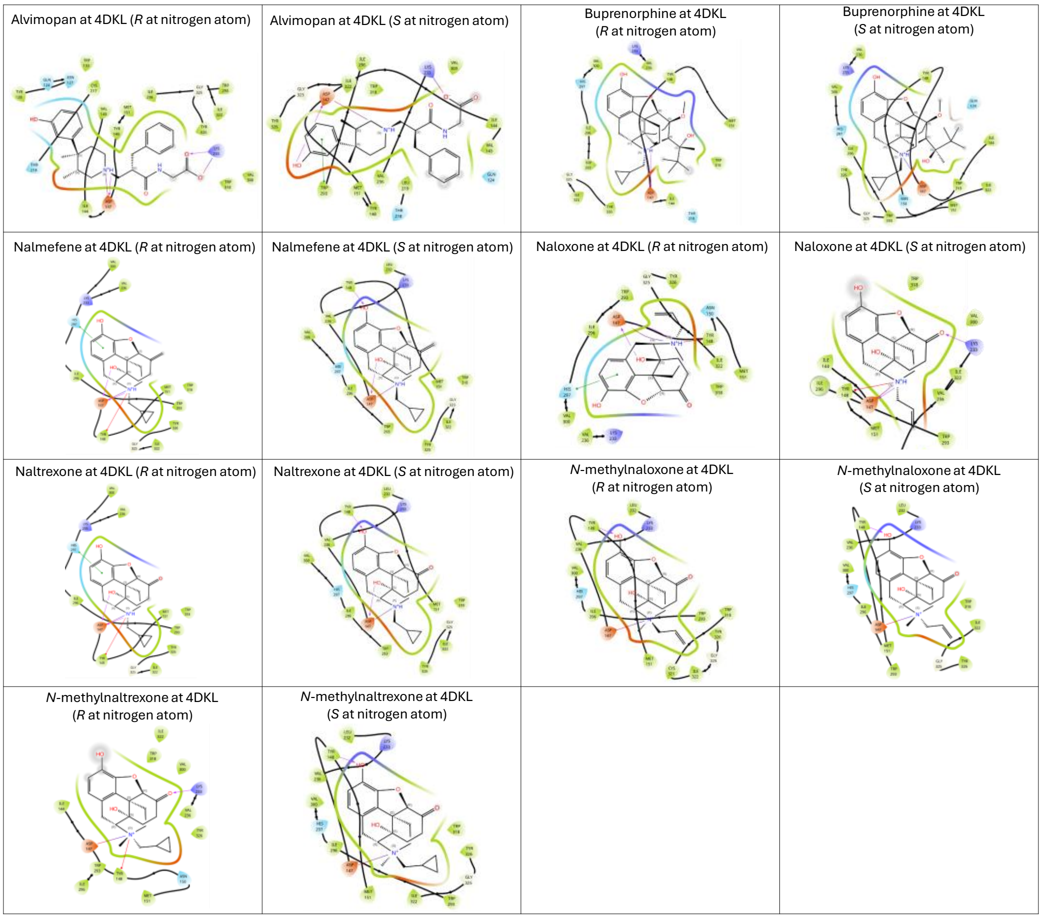

Figure S2. Interaction diagram between individual ligands and the opioid receptor (PDB ID: 4DKL) obtained from molecular docking.

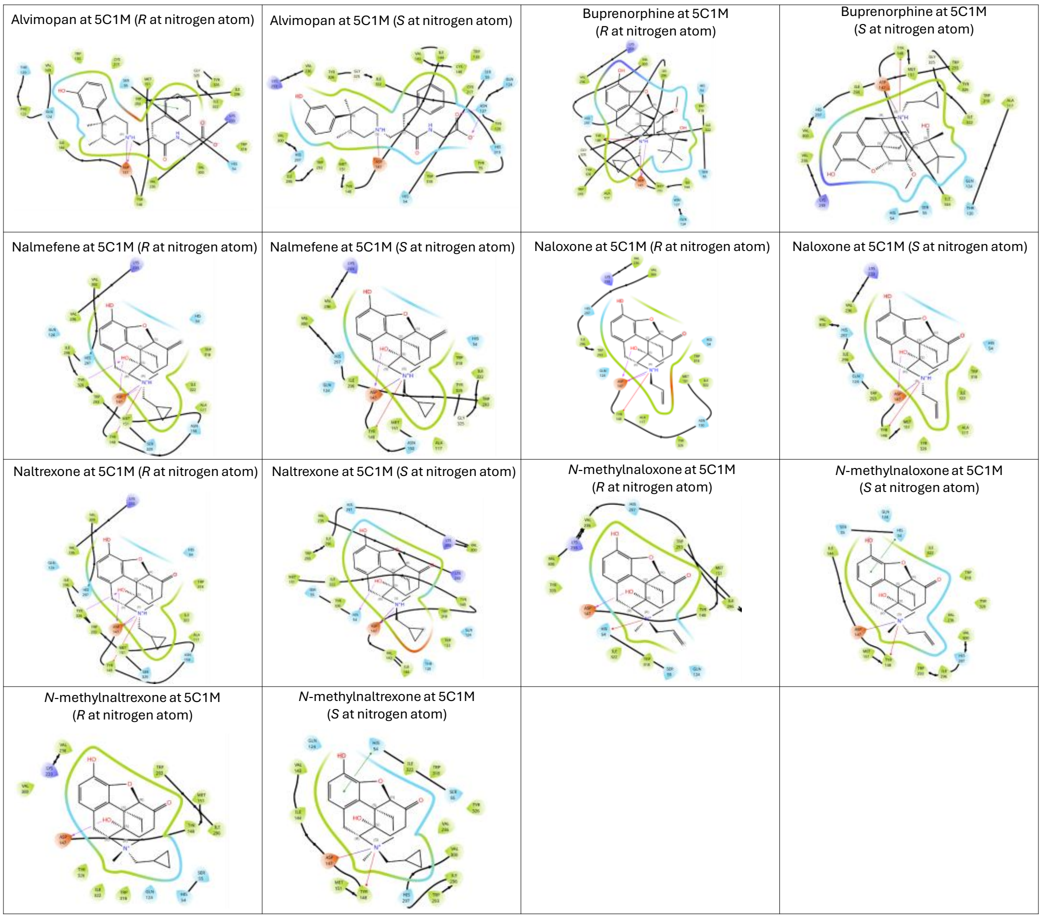

Figure S3. Interaction diagram between individual ligands and the opioid receptor (PDB ID: 5C1M) obtained from molecular docking.

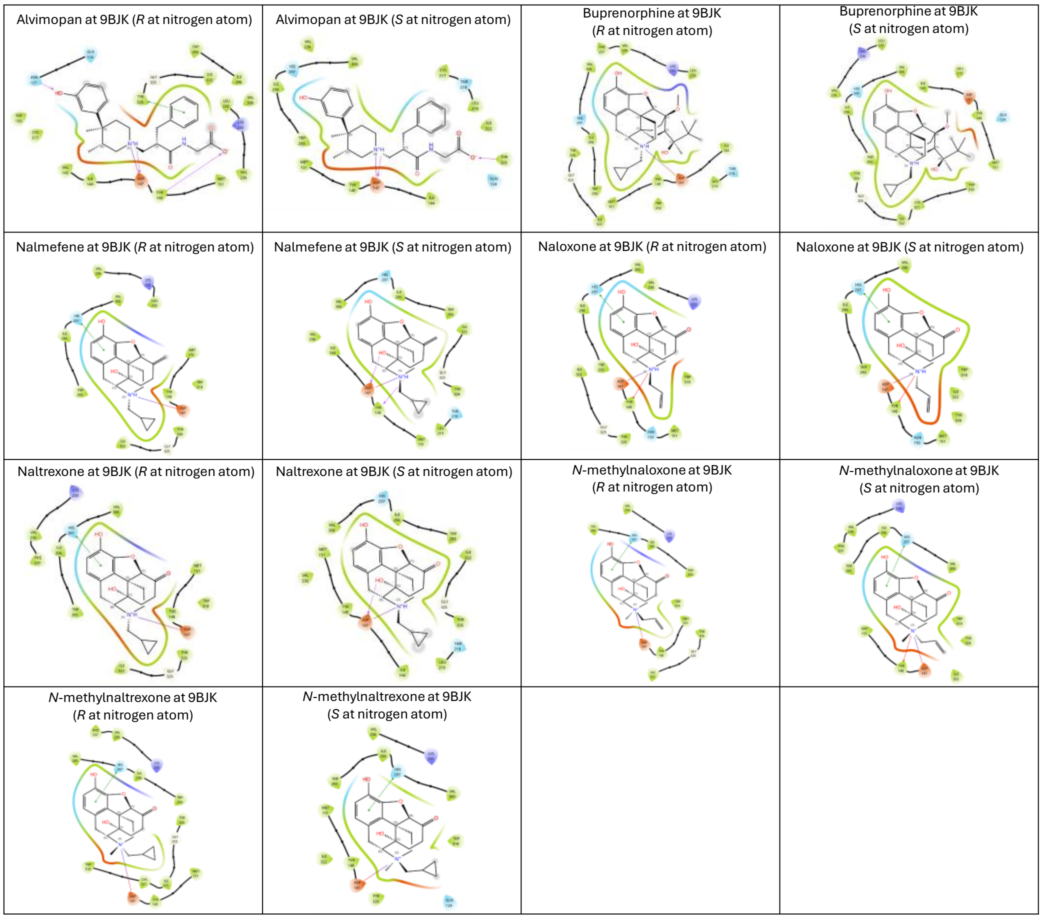

Figure S4. Interaction diagram between individual ligands and the opioid receptor (PDB ID: 9BJK) obtained from molecular docking.

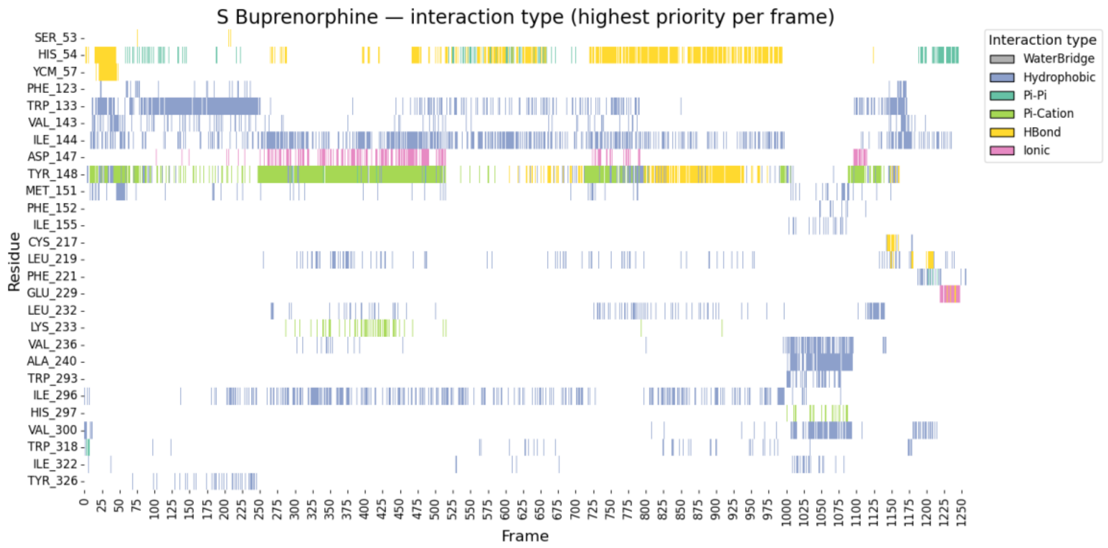

Figure S5.1. Time-resolved interaction map showing the dominant (highest-priority) interaction type for each frame in the RAMD trajectories of Buprenorphine.

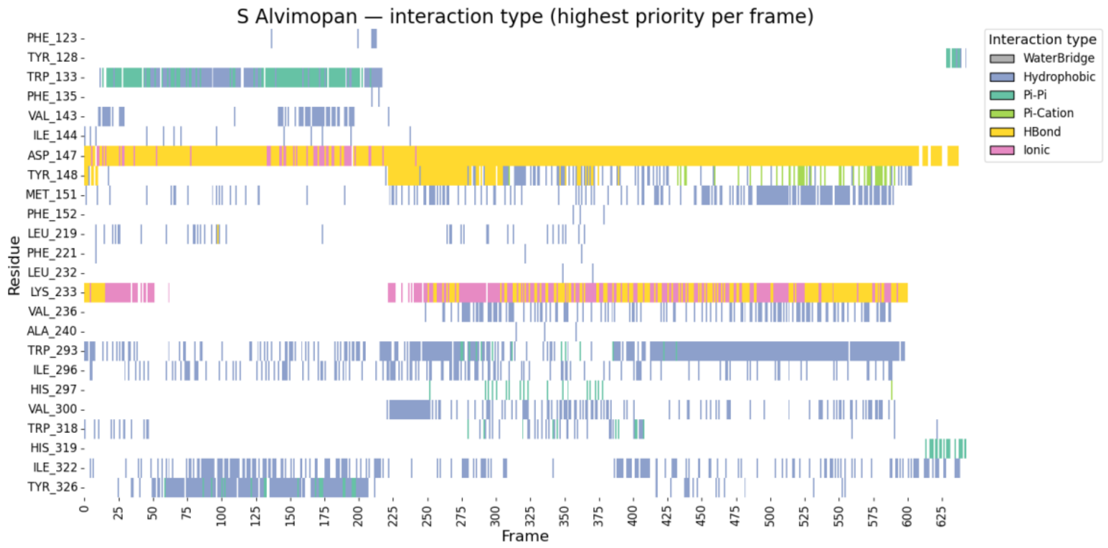

Figure S5.2. Time-resolved interaction map showing the dominant (highest-priority) interaction type for each frame in the trajectories of Alvimopan.

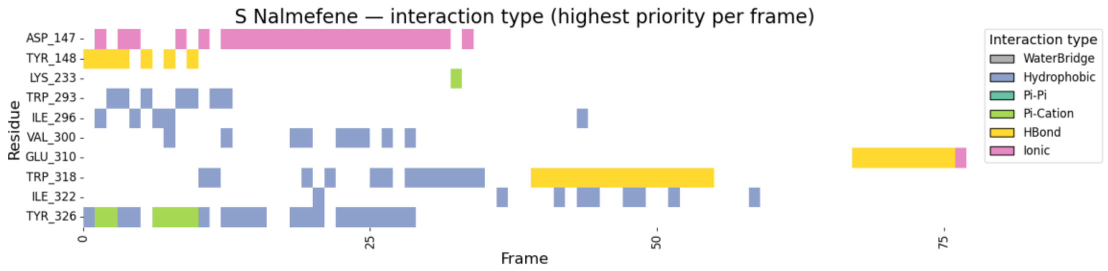

Figure S5.3. Time-resolved interaction map showing the dominant (highest-priority) interaction type for each frame in the RAMD trajectories of Nalmefene.

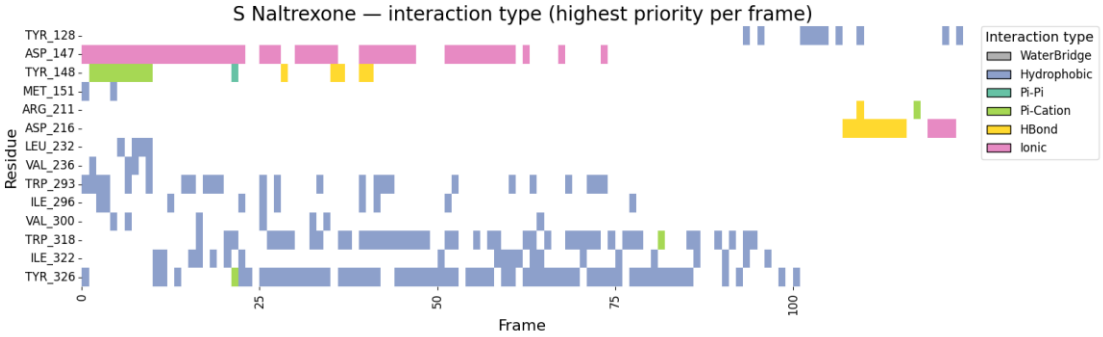

Figure S5.4. Time-resolved interaction map showing the dominant (highest-priority) interaction type for each frame in the RAMD trajectories of Naltrexone.

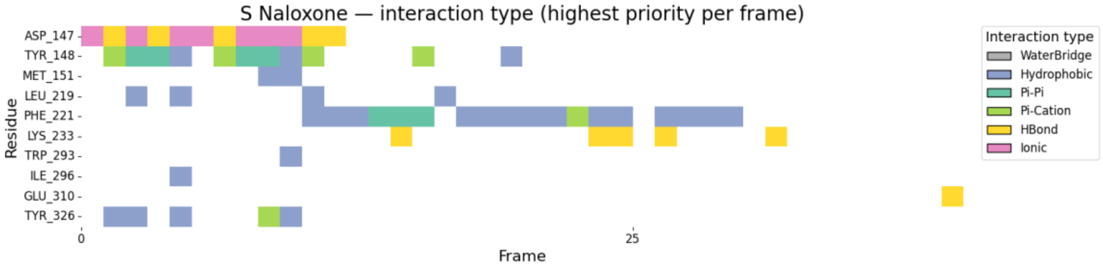

Figure S5.5. Time-resolved interaction map showing the dominant (highest-priority) interaction type for each frame in the RAMD trajectories of Naloxone.

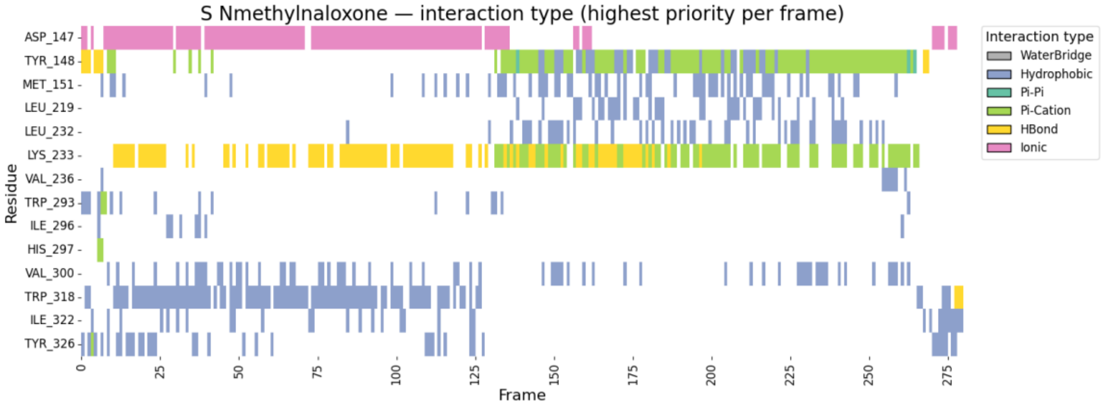

Figure S5.6. Time-resolved interaction map showing the dominant (highest-priority) interaction type for each frame in the RAMD trajectories of N-methylnaloxone.

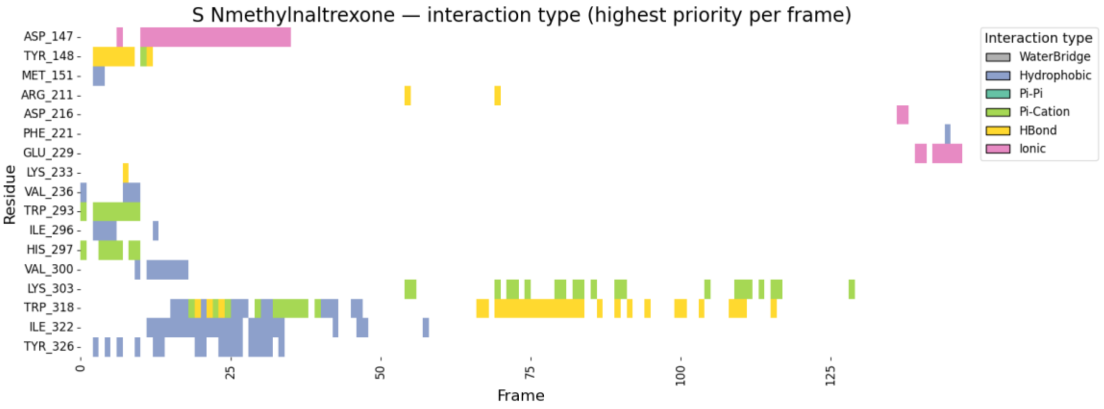

Figure S5.7. Time-resolved interaction map showing the dominant (highest-priority) interaction type for each frame in the RAMD trajectories of N-methylnaltrexone.

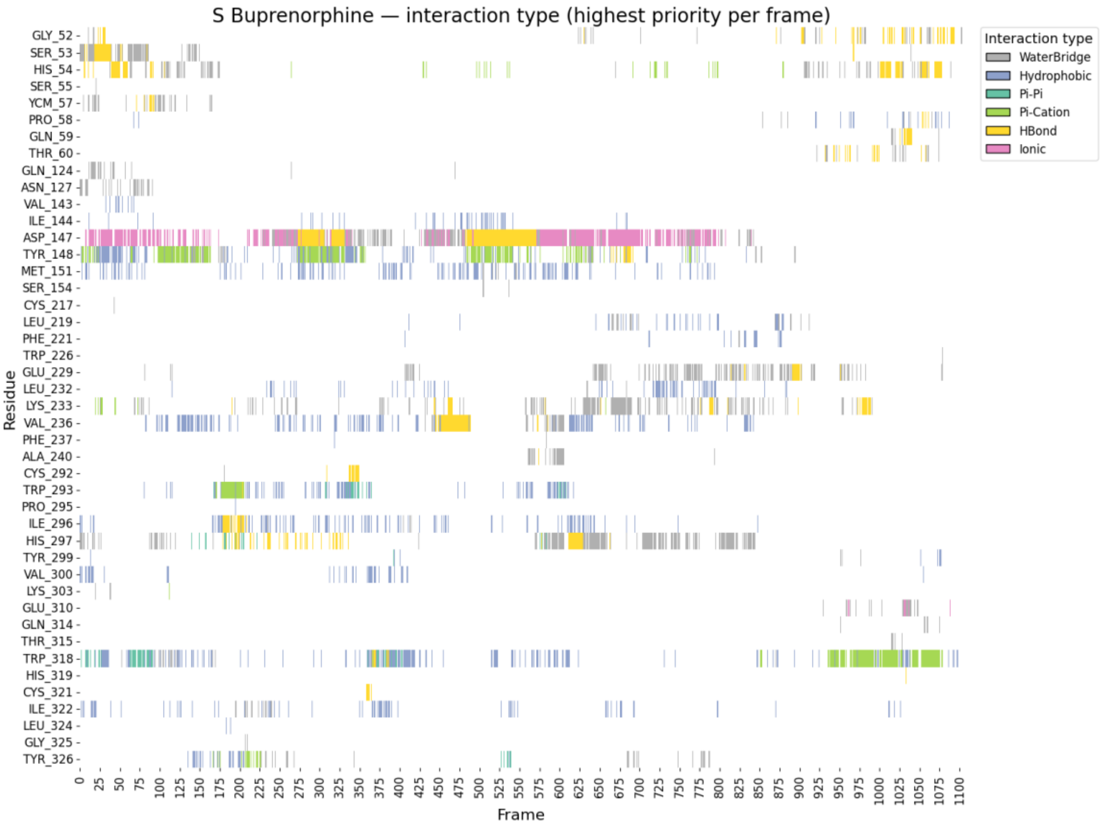

Figure S.6.1. Time-resolved interaction map showing the dominant (highest-priority) interaction type for each frame in the metaD trajectories of Buprenorphine.

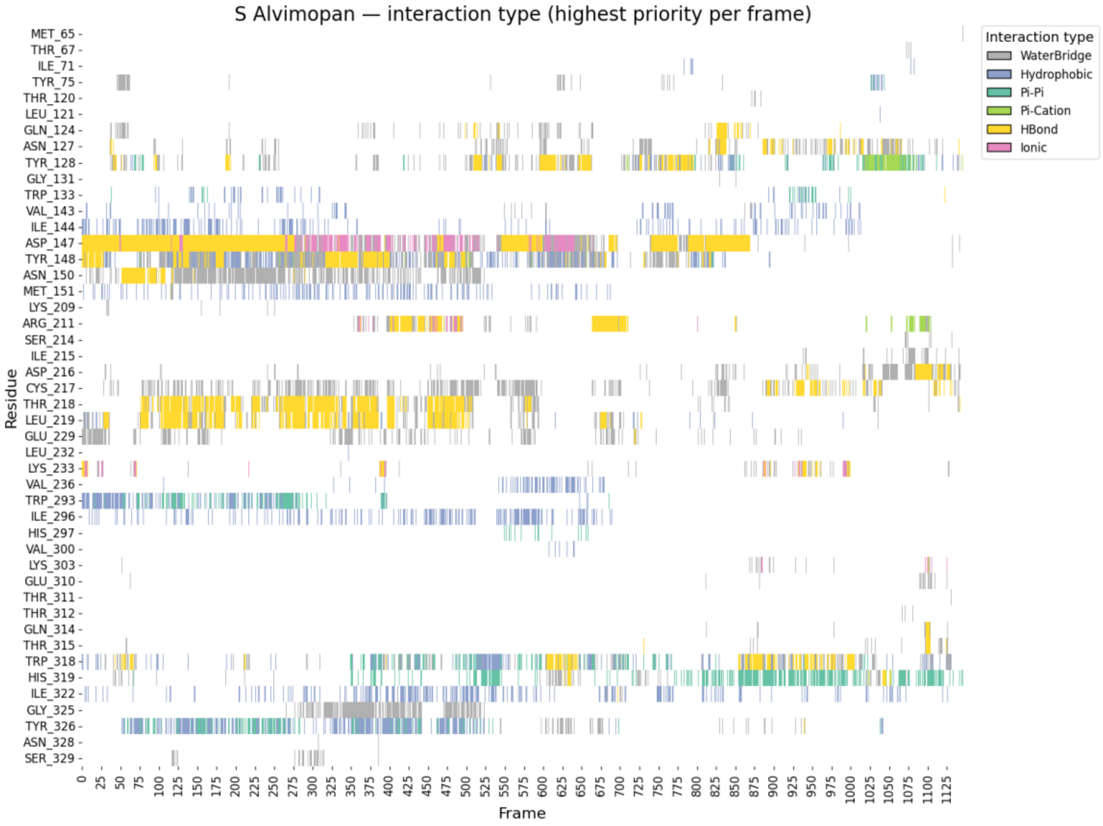

Figure S.6.2. Time-resolved interaction map showing the dominant (highest-priority) interaction type for each frame in the metaD trajectories of Alvimopan.

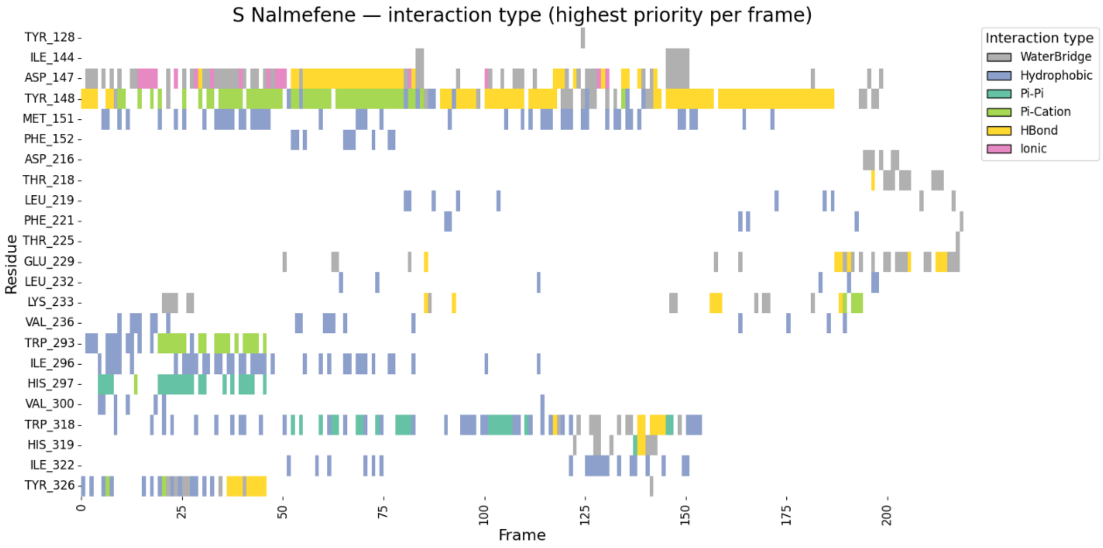

Figure S.6.3. Time-resolved interaction map showing the dominant (highest-priority) interaction type for each frame in the metaD trajectories of Nalmefene.

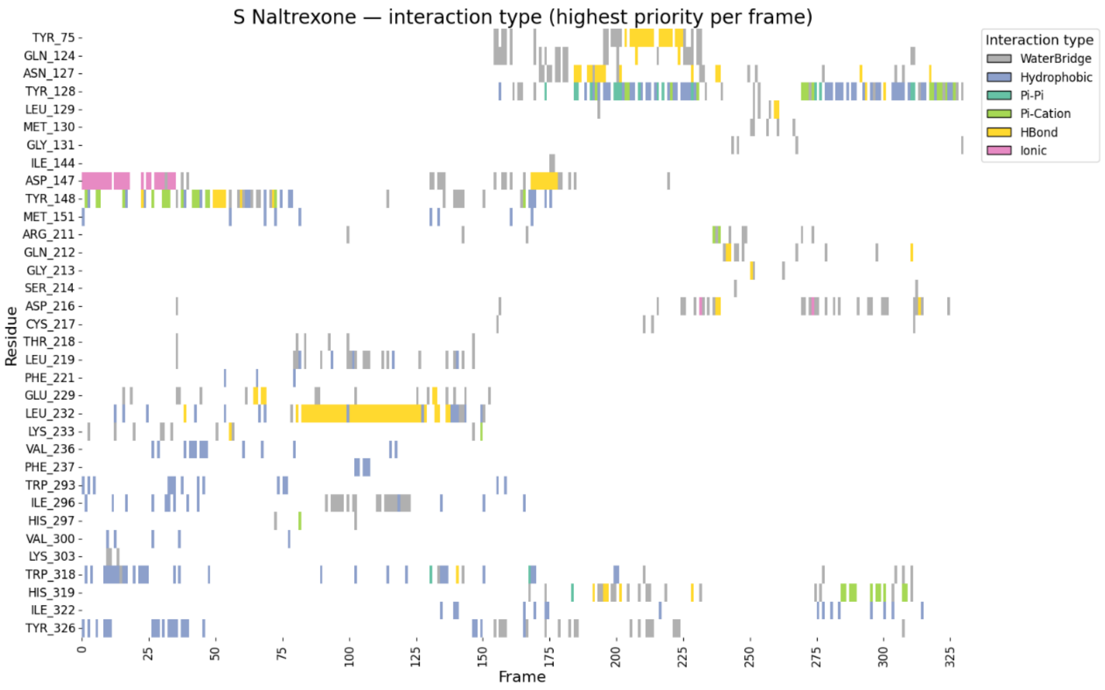

Figure S.6.4. Time-resolved interaction map showing the dominant (highest-priority) interaction type for each frame in the metaD trajectories of Naltrexone.

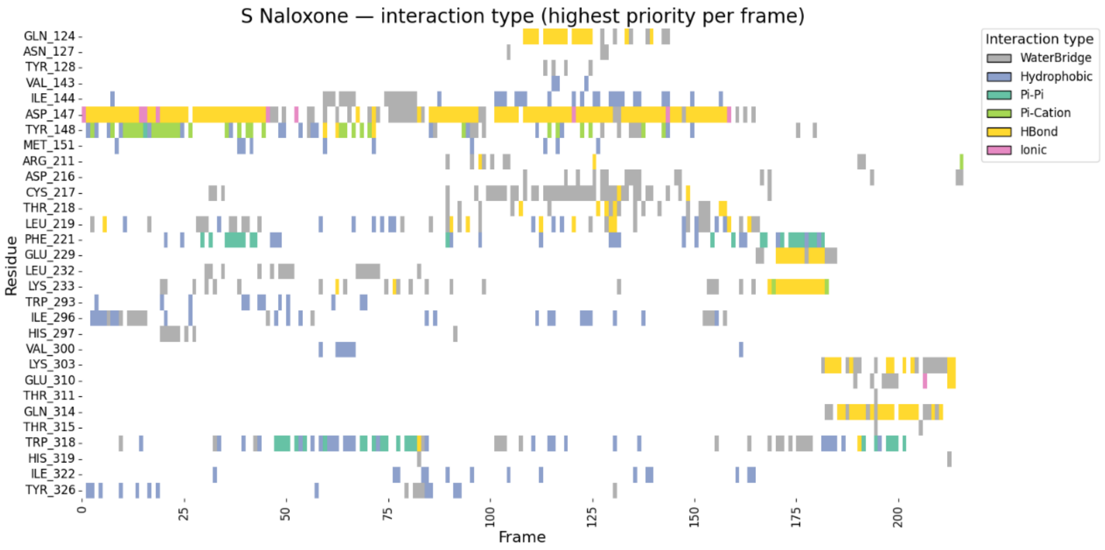

Figure S.6.5. Time-resolved interaction map showing the dominant (highest-priority) interaction type for each frame in the metaD trajectories of Naloxone.

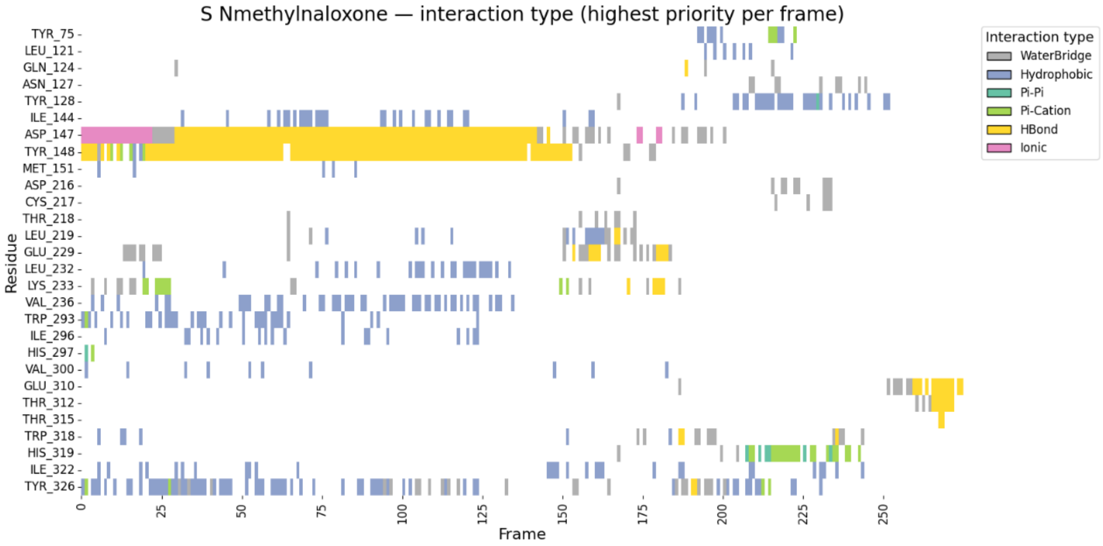

Figure S.6.6. Time-resolved interaction map showing the dominant (highest-priority) interaction type for each frame in the metaD trajectories of N-methylnaloxone.

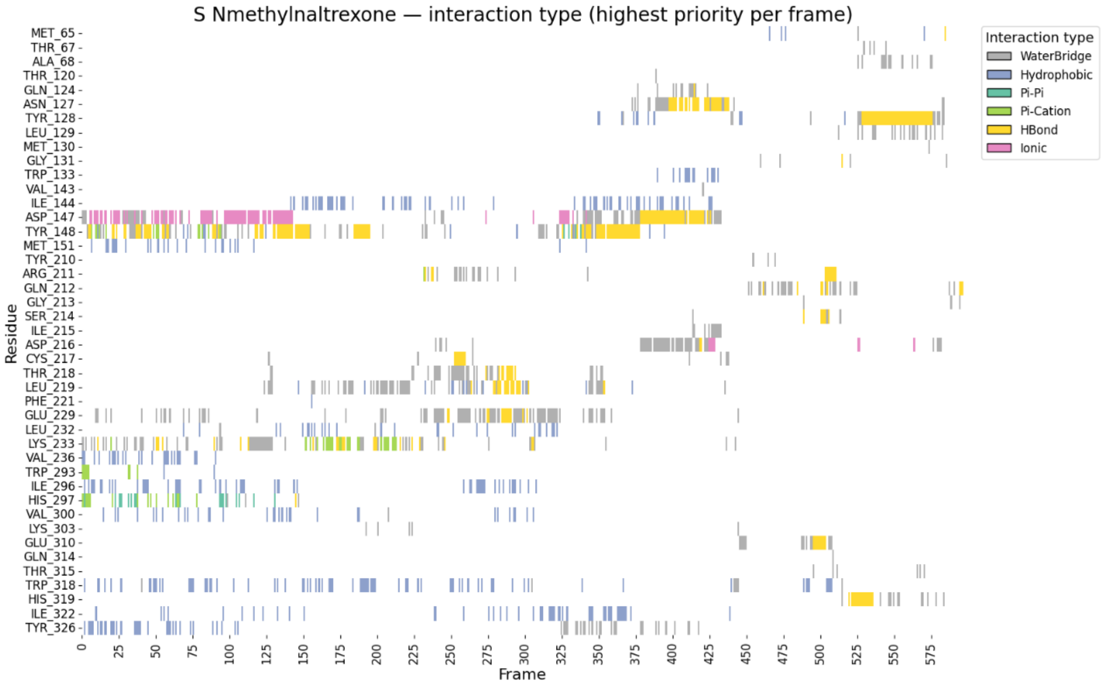

Figure S.6.7. Time-resolved interaction map showing the dominant (highest-priority) interaction type for each frame in the metaD trajectories of N-methylnaltrexone.

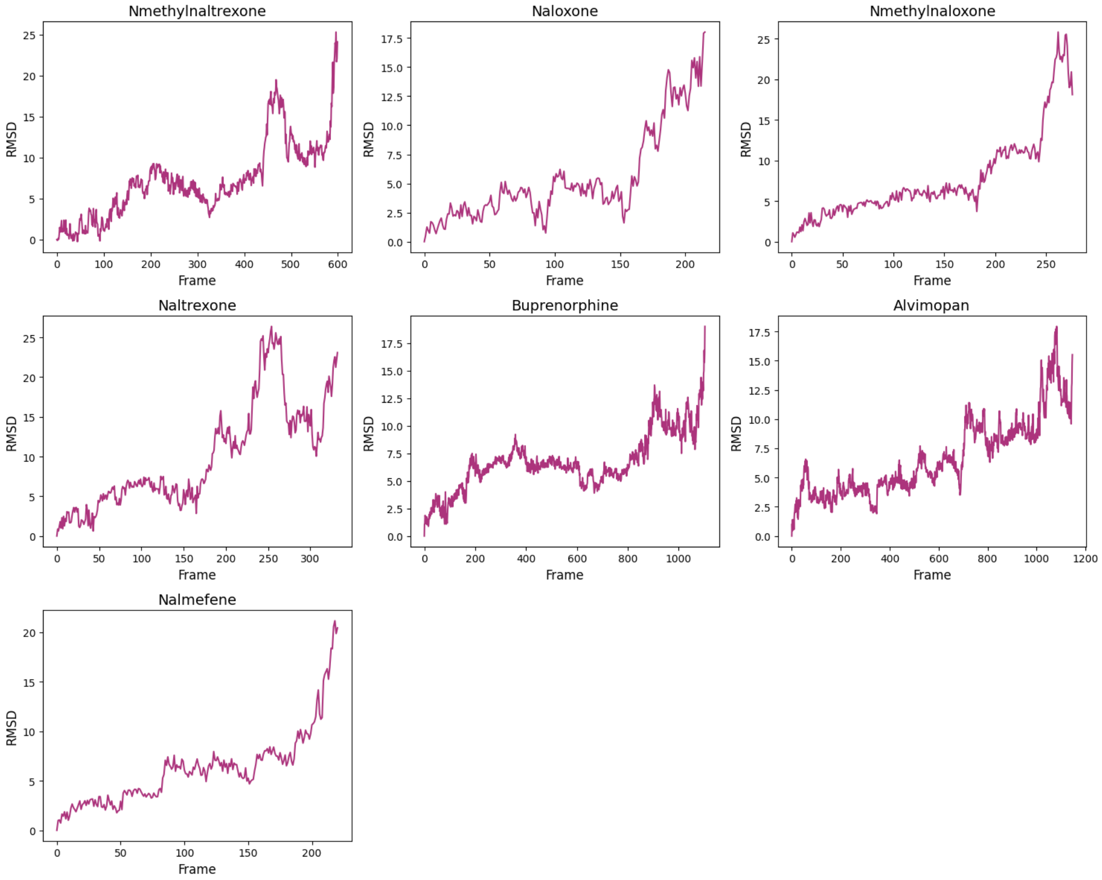

Figure S7. RMSD of ligand aligned on protein during MetaD trajectories.

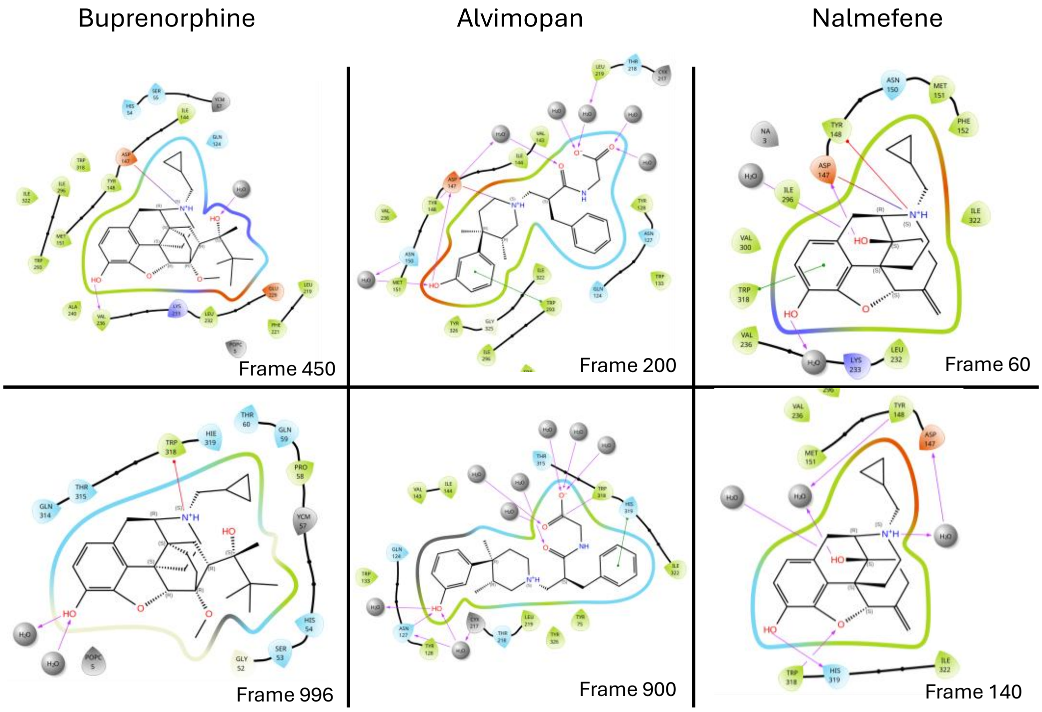

Figure S8. Ligand Interaction Diagrams of meta stable poses during metaD trajectories of Buprenorphine, Alvimopan and Nalmefene.

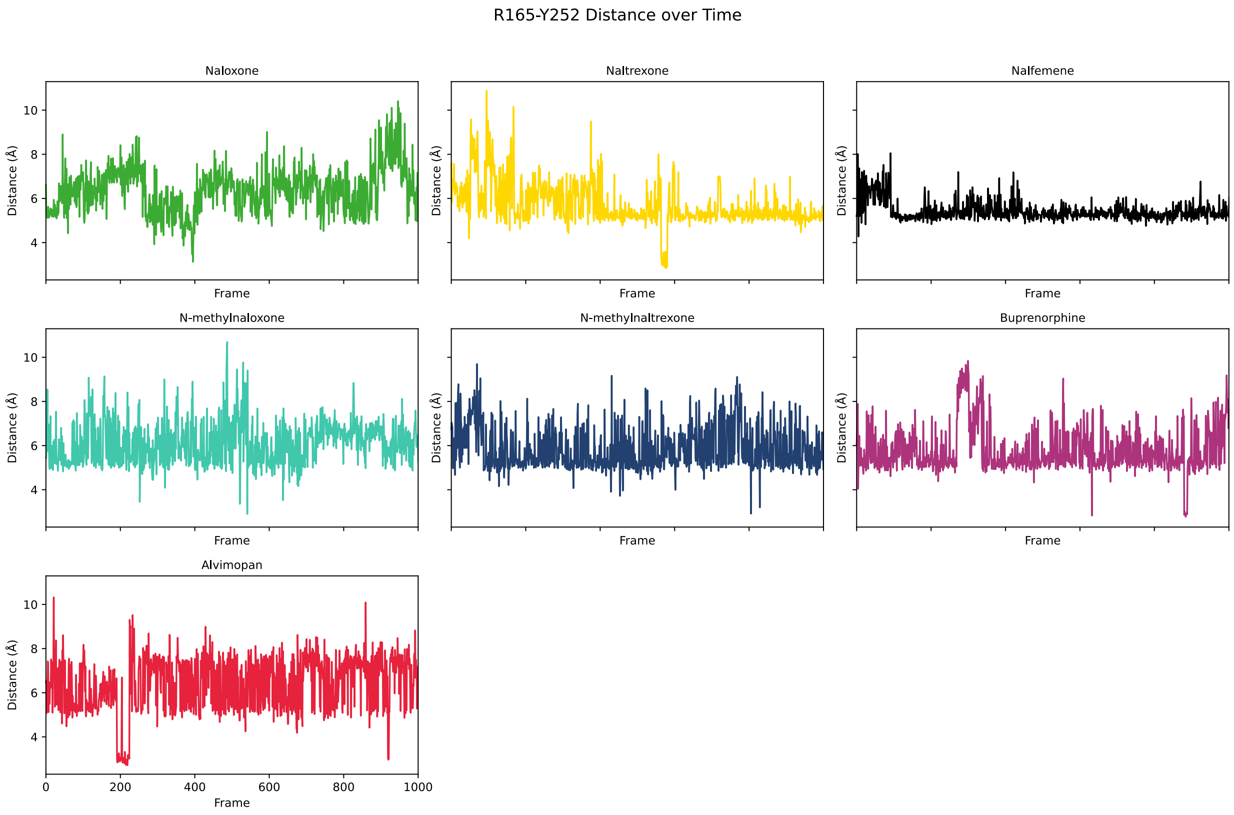

Figure S9. R165-Y252 distance in 5C1M crystal structure during MD.

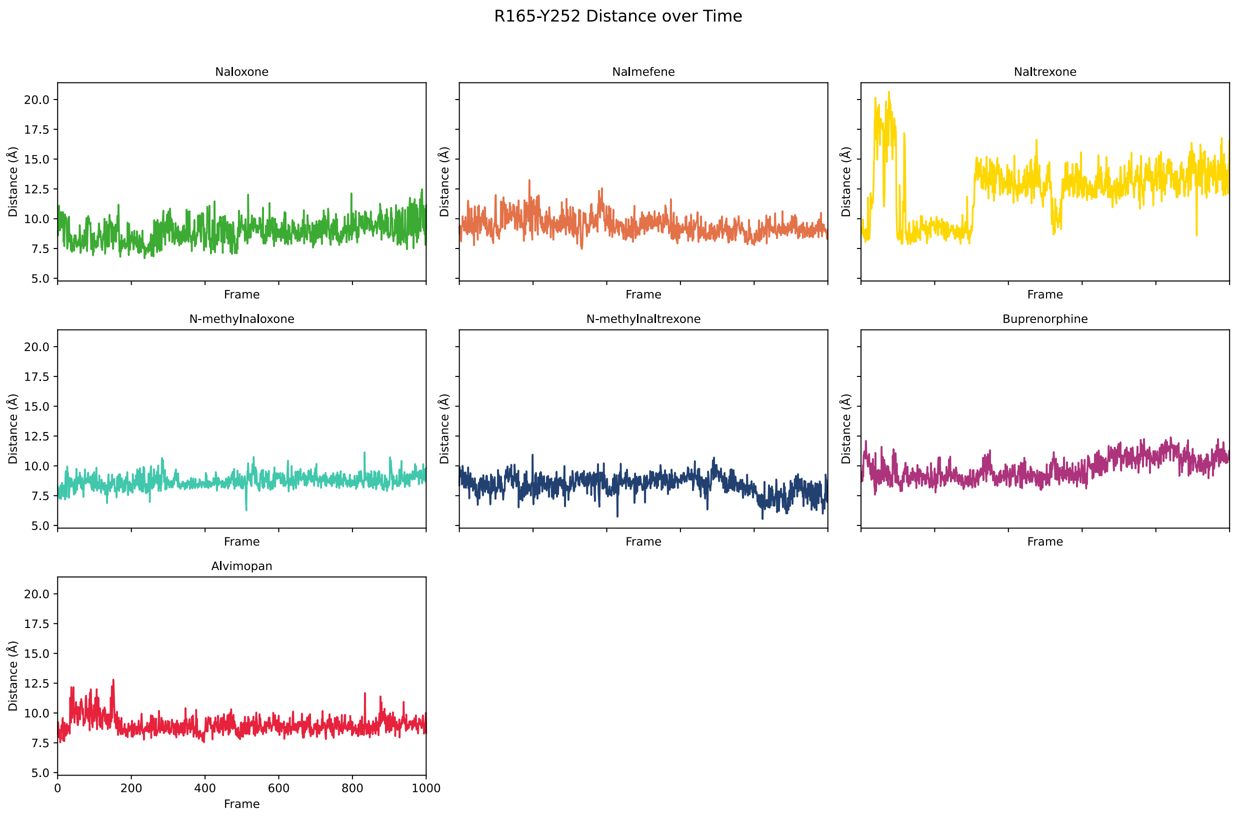

Figure S10. R165-Y252 distance in 9BJK crystal structure during MD.

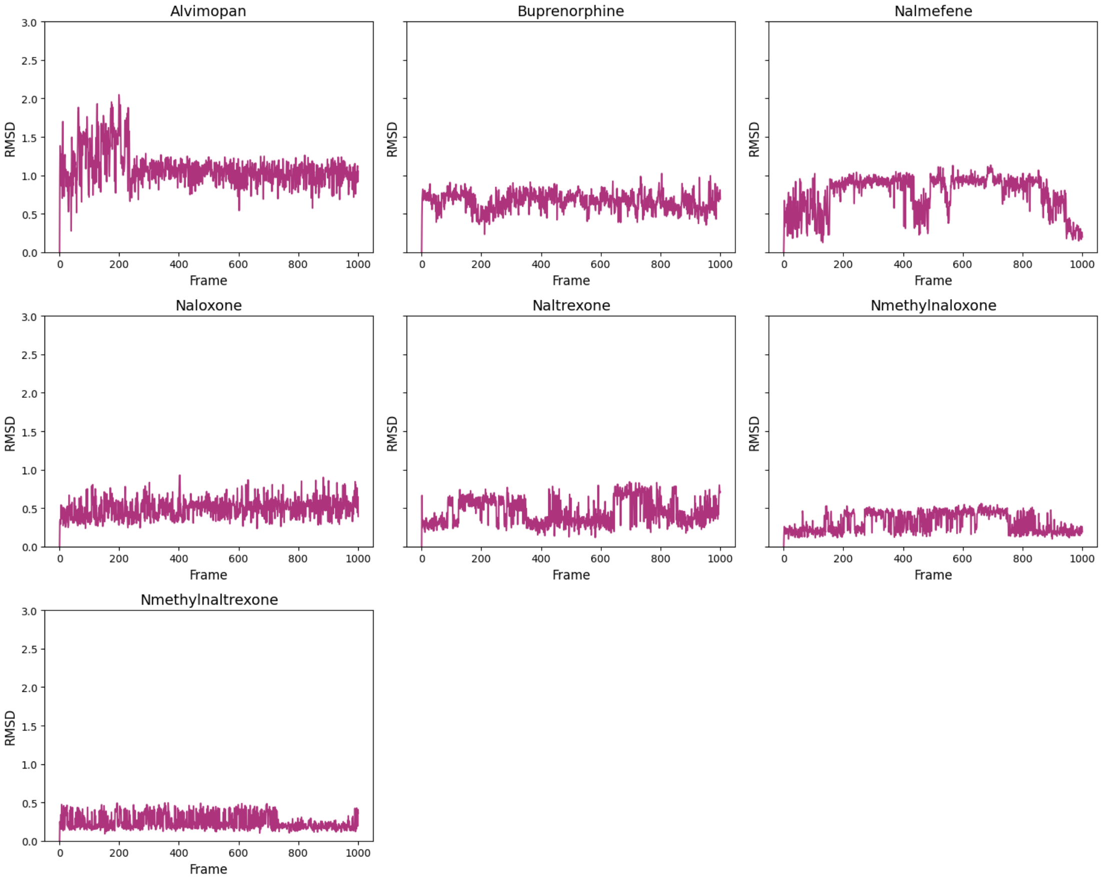

Figure S11.1. Ligand RMSD values relative to the initial docking poses monitored throughout the 2000 ns classical MD simulations for the 4DKL crystal structure. RMSD was calculated after protein backbone alignment to the initial frame.

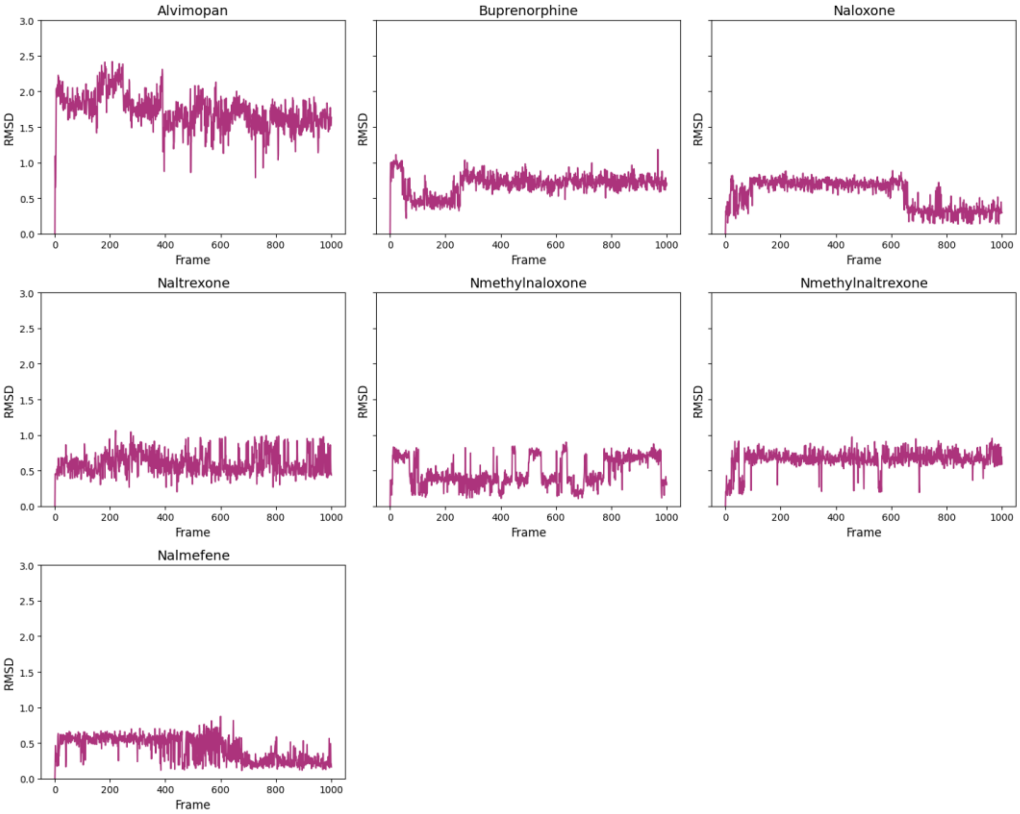

Figure S11.2. Ligand RMSD values relative to the initial docking poses monitored throughout the 2000 ns classical MD simulations for the 5C1M crystal structure. RMSD was calculated after protein backbone alignment to the initial frame.

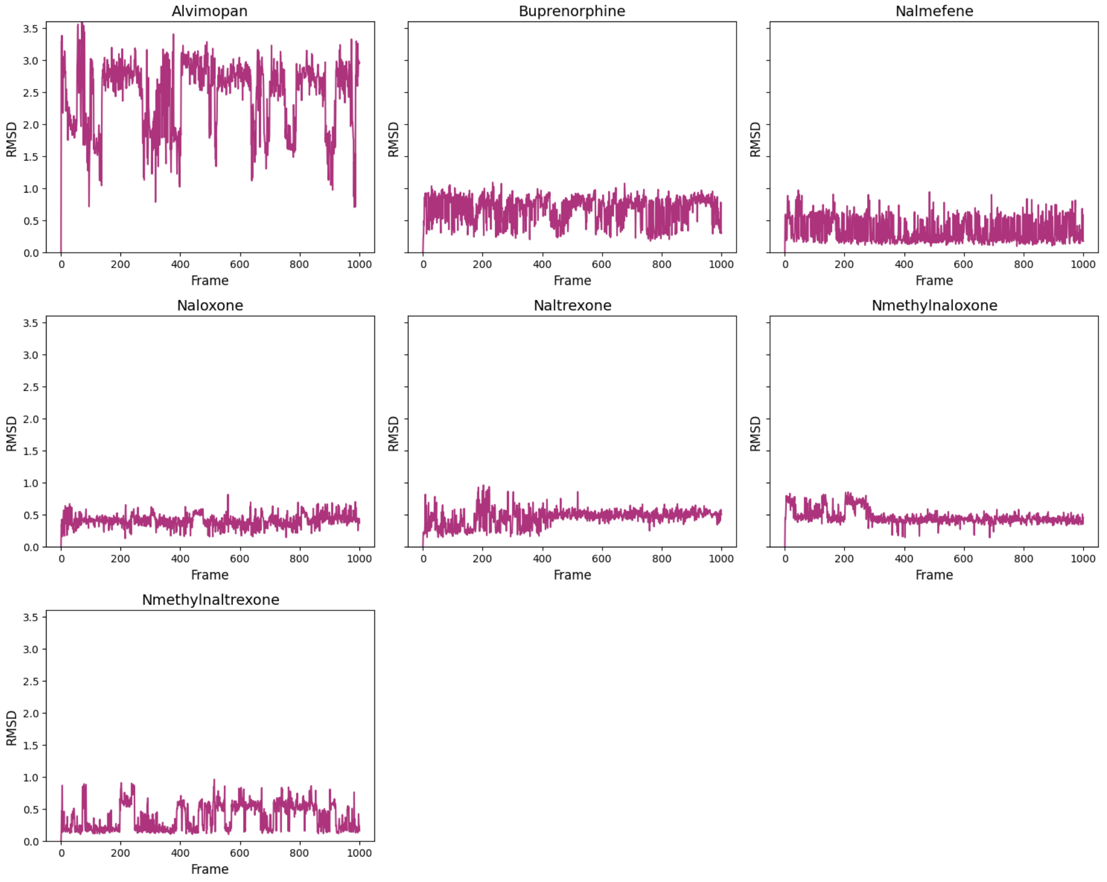

Figure S11.3. Ligand RMSD values relative to the initial docking poses monitored throughout the 2000 ns classical MD simulations for the 9BJK crystal structure. RMSD was calculated after protein backbone alignment to the initial frame.

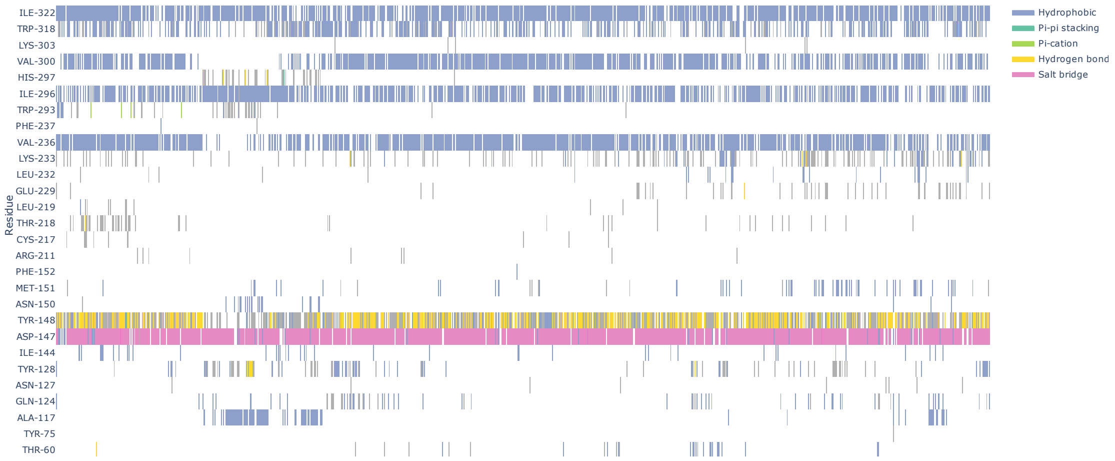

Figure S.12.1. Time-resolved interaction map showing the dominant (highest-priority) interaction type for each frame in the MD trajectory of Buprenorphine. Generated using CORAL-MD tool.

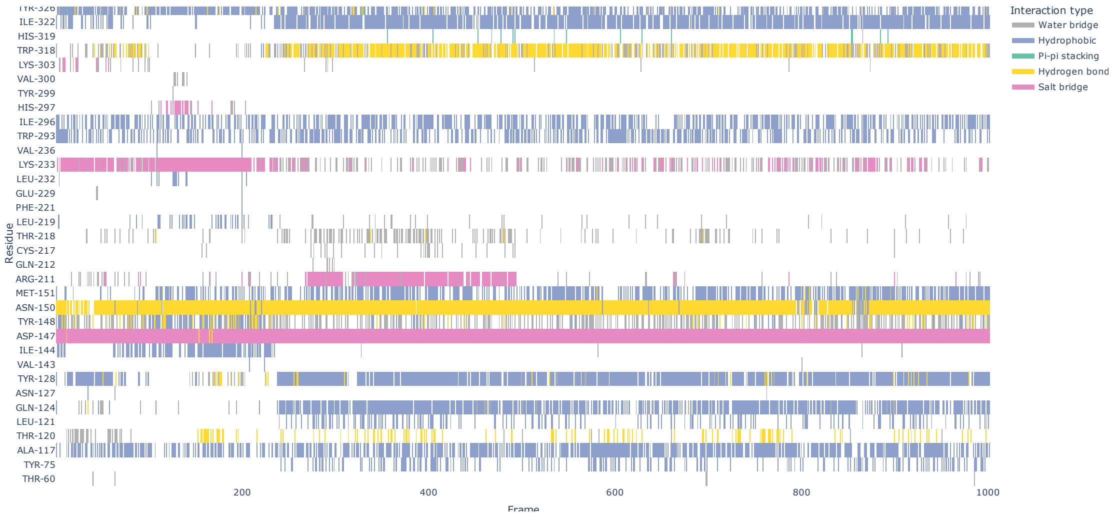

Figure S.12.2. Time-resolved interaction map showing the dominant (highest-priority) interaction type for each frame in the MD trajectory of Alvimopan. Generated using CORAL-MD tool.

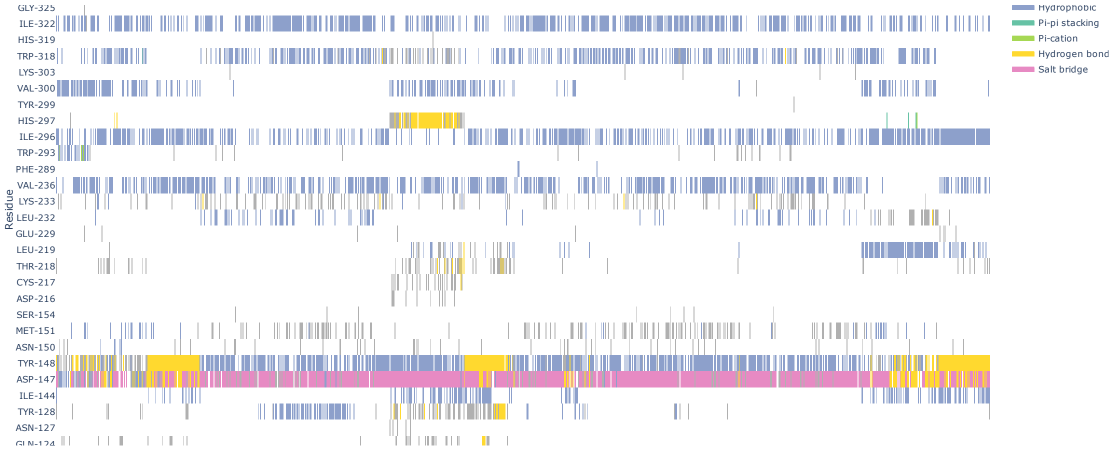

Figure S.12.3. Time-resolved interaction map showing the dominant (highest-priority) interaction type for each frame in the MD trajectory of Nalmefene. Generated using CORAL-MD tool.

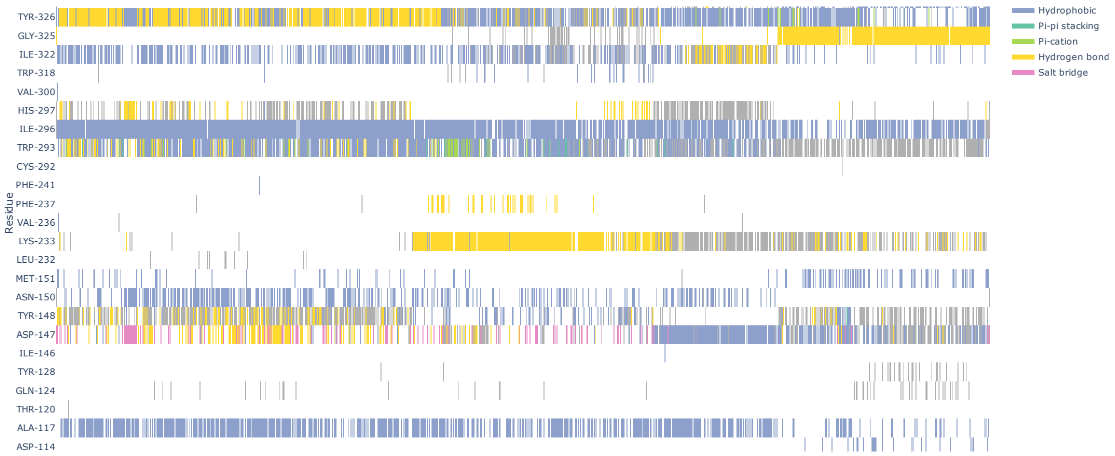

Figure S.12.4. Time-resolved interaction map showing the dominant (highest-priority) interaction type for each frame in the MD trajectory of Naltrexone. Generated using CORAL-MD tool.

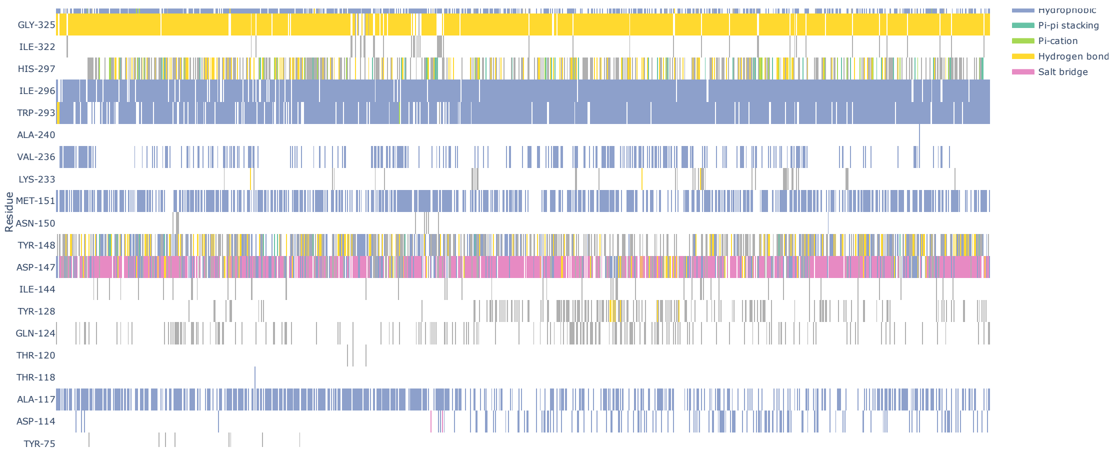

Figure S.12.5. Time-resolved interaction map showing the dominant (highest-priority) interaction type for each frame in the MD trajectory of Naloxone. Generated using CORAL-MD tool.

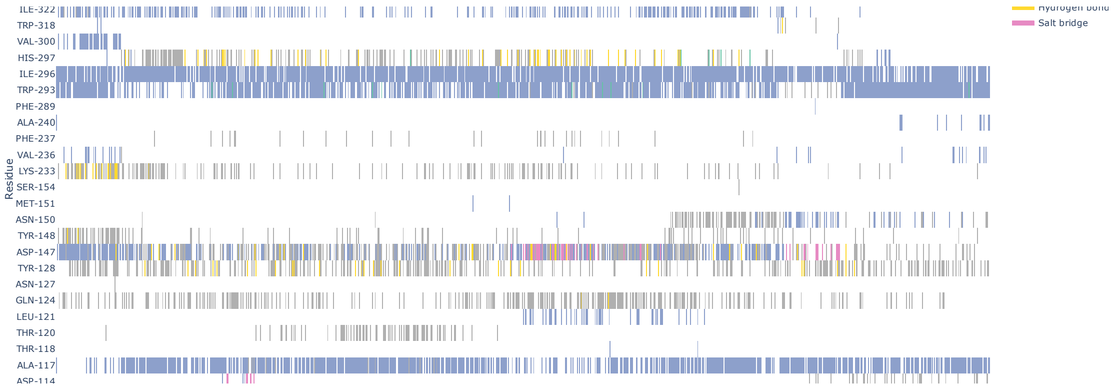

Figure S.12.6. Time-resolved interaction map showing the dominant (highest-priority) interaction type for each frame in the MD trajectory of N-methylnaloxone. Generated using CORAL-MD tool.

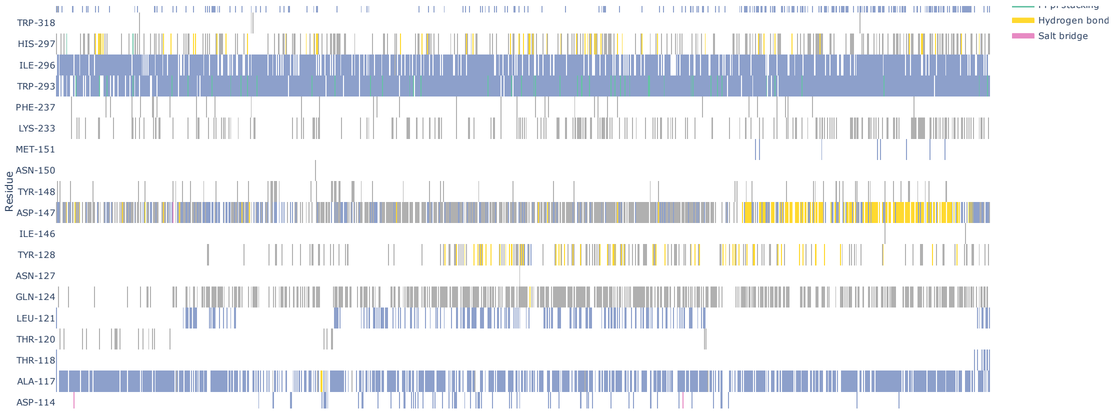

Figure S.12.7. Time-resolved interaction map showing the dominant (highest-priority) interaction type for each frame in the MD trajectory of N-methylnaltrexone. Generated using CORAL-MD tool.
